# The Relationship Between the Functional Integration and Topological Complexity of Human Electroencephalographic (EEG) Signals: Three Moderating Factors

**DOI:** 10.3390/brainsci16080796

**Published:** 2026-07-28

**Authors:** Logan T. Trujillo

**Affiliations:** Department of Psychology, Texas State University, San Marcos, TX 78666, USA; logant@txstate.edu; Tel.: +1-512-245-3623

**Keywords:** functional topological complexity, functional integration, EEG, volume conduction, regularity, modularity

## Abstract

**Highlights:**

**What are the main findings?**

- EEG-based functional integration and topological complexity follow a curvilinear inverted-U relationship at the level of the cortex and an inverse relationship at the level of the scalp.
- This discrepancy occurs for irregular (aperiodic) but not regular (periodic) cortical EEG source signals and is not the result of EEG source modularity.

**What are the implications of the main findings?**

- The spatial mixing of volume-conducted cortical EEG signals alters the functional integration and topological complexity of scalp EEG signals because it smooths scalp EEG irregularity.
- Future studies of the brain’s functional organization must take this factor into account when interpreting the functional integration and topological complexity of the brain as measured using scalp EEG.

**Abstract:**

**Background and Objectives**: A useful strategy to study the dynamic functioning of the human brain is to focus on the relationship between the brain’s functional integration and topological complexity. Theoretical analysis using information-theoretic metrics shows that the functional topological complexity of the brain has a curvilinear inverted-U relationship with its functional integration. This relationship is supported by simulation studies of the functional integration and topological complexity of cortical electroencephalographic (EEG) dipole sources. However, scalp-level EEG functional topological complexity follows an inverse relationship with functional integration. The present study investigated this discrepancy for the case of alpha-range (7–13 Hz) EEG signals predominant during wakeful resting brain states. **Methods**: Three different EEG source factors were explored that could affect the integration–complexity relationship for scalp-level EEG: volume conduction, regularity, and modularity. The EEG simulations parametrically manipulated the regularity and modularity of cortical EEG dipole source signals and their scalp-level projections to identify how EEG functional integration and topological complexity depended on these factors at the cortical and scalp levels before and after the spatial mixing effects of volume conduction. Simulation findings were compared with results obtained from empirically measured resting state EEG signals. **Results**: Simulated source-level EEG functional integration and topological complexity followed a curvilinear inverted-U relationship, whereas scalp-level functional topological complexity had an inverse relationship with functional integration, with this difference occurring for irregular (aperiodic) sources but not regular (periodic) sources. EEG source modularity was related to the functional integration–complexity relationship only for regular EEG signals. These findings were consistent with the results obtained from the empirical EEG signals. **Conclusions**: The discrepancy between source- and scalp-level EEG functional integration–complexity relationships primarily reflects the spatial mixing effects of volume conduction and its secondary effects on scalp EEG regularity.

## 1. Introduction

### 1.1. The Functional Organization of the Brain

It is widely accepted that the dynamic functioning of the human brain reflects the interactions of diverse neuronal networks across local and global spatiotemporal scales. Specialized information processing is segregated at local network scales, whereas global scale networks integrate local network activity into a unified brain-wide information process [1,2,3,4,5,6,7]. Importantly, the brain as a system is highly complex because its segregation and integration adaptively evolve to form dynamic, interconnected neuronal hierarchies [4]. The complexity of the brain and its associated segregation and integration have been shown to be crucial for human cognition, behavior, mental health, and consciousness [8,9,10,11,12,13,14]. Thus, a better understanding of these organizational properties of the brain will inform interpretations of their functional relevance.

### 1.2. The Relationship Between the Brain’s Functional Integration and Complexity

One useful strategy to study the complexity, integration, and segregation of the brain is to focus on the relationship between integration and complexity, because this implicitly captures the segregation of the brain as well (integration and segregation have an inverse relationship to each other; so, when integration is low, segregation is high and vice-versa). The integration and complexity of the brain may be quantified in many ways, with the specific approach dependent on how one conceptualizes the brain’s complexity [4]. Complexity can reflect the evolution of a system’s states or the repetitiveness of a system’s trajectory through its state space [15], with these distinctions manifesting in temporal or topological dimensions [16,17]. However, an influential way of conceptualizing the brain’s complexity is as an intermediate state between randomness and order [3,4,17,18,19]. Most research on this kind of complexity has focused on its topological aspects, which reflect the dynamic interplay between the functional segregation and integration within a neural system via interactions among its neurons [4,6,7,17]. Such brain complexity is the focus of this paper, where it is called functional topological complexity or by the abbreviated terms functional complexity (following [17]), topological complexity, or simply complexity.

Functional topological complexity and corresponding functional integration may be quantified in various ways; the present study utilized quantification techniques based on concepts from information theory [4,6,7,20,21,22]. In this information-theoretic framework, the brain’s functional integration is quantified in terms of “deviations from statistical independence among components of a neural system” [6] (p. 5033). The brain’s functional topological complexity is quantified in this framework via a metric called neural complexity, in which a neural system is partitioned into subsets composed of increasing numbers of neuronal elements (subset size). Neural complexity is then quantified as the area between the linear increase of integration and the average integration as functions of subset size. Calculating the neural complexity for large systems can be computationally intensive, so it is often useful to use a simpler proxy measure instead. One such proxy measure is the interaction complexity, which indexes the information content resulting from the interactions among the brain’s elements [4].

**Figure 1:**
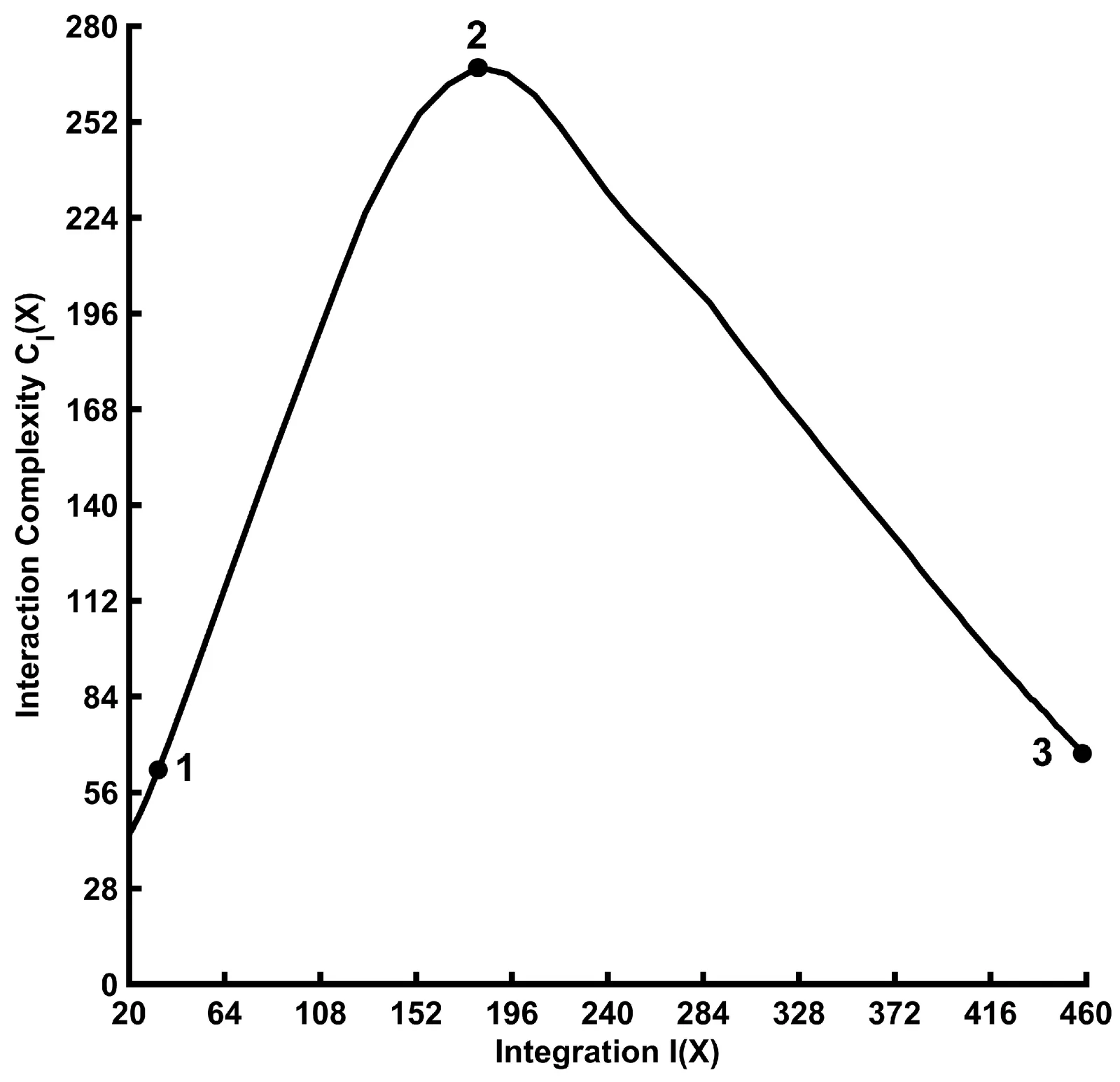
The theoretical relationship between functional integration, *I*(*X*), and functional topological complexity as indexed by the interaction complexity metric, *C_I_*(*X*); see Section 2.7: Computation of EEG Functional Integration and Topological Complexity, below, for details of the mathematical notation and computational methods. In this example, *C_I_*(*X*) was computed for 36 zero-mean multivariate Gaussian time courses using Toeplitz covariance matrices with increasing intervariable dependencies. The curve reflects the mean across 10 separate simulations (1 s trials; 100 trials per simulation); values are in units of bits of information. The points 1, 2, and 3 on the curve exemplify increasing levels of multivariate statistical dependence and integration; for further description, see text of Section 1.2. Figure adapted from [23] with permission of the author.

There is a curvilinear relationship between functional integration and topological complexity that approximately follows an inverted-U shaped function [6]; Figure 1 illustrates this relationship for the case of interaction complexity. Functional complexity is initially small at low integration values when the brain’s components are statistically independent and then increases to a maximum as integration increases from low to intermediate levels when there is heterogenous statistical dependence among the brain’s components (i.e., when a system is a mixture of integrated and segregated; [4,6]). However, functional complexity decreases as integration increases from intermediate to high values when the brain’s components are fully statistically dependent.

The main topic of this paper is the nature of this functional integration–complexity relationship as quantified from the brain’s electrical signals measured using electroencephalography (EEG). Several previous studies have examined the theoretical relationship between functional integration and topological complexity (or its proxies) as applied to simulated and empirical EEG brain signals [4,6,23,24]. One key conclusion that can be drawn from this research is that the functional integration and topological complexity of cortical EEG dipole sources follow the theoretical inverted-U relationship, but scalp EEG signals inhabit the medium- to high-integration regime on the right side of peak of the complexity curve in Figure 1, where topological complexity is a decreasing function of integration. This latter observation was initially reported by [24] (and replicated by [23]), who found that simulated dipole sources followed the inverted-U relationship, whereas the simulated scalp EEG did not. Instead, the corresponding scalp-level EEG functional topological complexity largely decreased as functional integration increased over low, medium, and high EEG integration levels. The empirical scalp EEG signals demonstrated a similar monotonically decreasing relationship between the functional integration and topological complexity of the EEG signals, even when including surrogate EEG data created by randomizing the phase relationships among the EEG sources of the empirical data. The study authors of [24] speculated that this discrepancy between the source-level and scalp level integration–complexity relationship may be due to an interaction between the spatial mixing of the source signals during their volume conduction from the brain to the scalp [25] and the periodic regularity of oscillatory EEG signals. This speculation was based on the observation that the discrepancy between the source- and scalp-level relationships occurred during an initial transition from aperiodic random noise dipole sources to periodic oscillatory dipole sources.

### 1.3. The Present Study

The goal of the present study was to understand this puzzling behavior of the EEG functional integration and topological complexity relationship at the scalp level of measurement relative to the integration–complexity relationship observed at the cortical source level. Given that most EEG studies of the brain’s functional organization employ scalp-level recordings, addressing this issue may assist the interpretation of scalp level EEG functional integration and topological complexity measures when they are used to study neurocognitive information processing. Many different factors can affect the measurement and quantification of EEG functional integration and topological complexity, including EEG recording parameters, length of time series, signal-to-noise ratios, biophysical properties of head and brain tissue, task instructions, and cognitive state [15,24,26]. The present study considered three specific factors. The first factor was the mixing effects of volume conduction, which was considered based on the observations of [23,24], as discussed earlier. The second factor was EEG signal regularity, which was considered based on the speculation of [24] and is rooted in the fact that neural complexity reflects the relative balance between random, irregular processes and perfectly ordered brain processes [4,6]. The oscillatory neural processes detectable by EEG have an intrinsic non-random autocorrelative structure that cannot be broken down further without changing the regularity (e.g., periodicity) of the signals. This suggests that EEG signals operate at integration levels corresponding to the right side of the peak of the complexity curve in Figure 1, with this behavior modulated by the mixing effects of volume conduction.

The third factor considered in the present study was the degree of segregation of the brain as indexed via a statistical metric called modularity, developed in brain network analysis [27]. The latter uses graphs to treat the brain as an interconnected system of nodes that represent distinct brain areas; structural or functional connections among these brain areas are represented by edges that connect the nodes [27]. Brain network nodes can be divided into different partitions based on specific patterns of connectivity among the nodes. Network communities or “modules” are subdivisions of densely connected network nodes that “are more strongly connected with each other than with other parts of the network” [27] (p. 303). Modularity and related network analysis measures (see Section 2.8: Estimation of Brain Network Organization, below) explicate this community structure, the latter of which is a specific realization of the way the brain may segregate or integrate its neuronal elements. Recent research suggests that brain modularity may be an important driver of global brain network dynamics and brain integration–segregation balance [3,17]. Thus, brain modularity metrics should provide good independent metrics to use to understand the behavior of EEG functional integration and topological complexity. (Note that in this manuscript, the term “modularity” is generally used to denote modular brain network community structure, whereas the specific network analysis statistic called “modularity” that quantifies the strength of network modularity is referred to using the standard mathematical notation for this quantity in the brain network analysis literature; see Section 2.8: Estimation of Brain Network Organization, below).

The present study assesses the relationship of these three factors to empirical and simulated EEG functional integration and topological complexity during the ongoing wakeful resting state of the brain. Following previous studies [23,24,26,28], analysis focuses on alpha-range EEG oscillations, which are predominant during wakeful resting states [29] and provide a robust starting point for EEG functional complexity analysis [26]. This choice was also motivated by evidence that information-theoretic complexity measures are more accurate when performed on data within a limited frequency range [28]. First, regularity, modularity, integration, and topological complexity were quantified from empirically measured resting state alpha-range EEG signals (see Section 3.1. Observed EEG Data, below). Then, EEG simulations were conducted that parametrically manipulated the regularity and modularity of cortical EEG sources and the corresponding scalp-level EEG signals (see Section 3.2: Dipole Simulation of Resting State EEG, below). This enabled identification of the relationship of EEG regularity and modularity to EEG functional integration and topological complexity both at the cortical source level and the scalp level after the spatial mixing effects of volume conduction. In turn, this provided insight into how these factors governed the relationship between scalp EEG functional integration and topological complexity. The results of these simulations were then used to interpret the regularity, modularity, and functional integration–complexity relationship of the empirically measured resting state alpha-range EEG signals (see Section 4: Discussion, below).

## 2. Materials and Methods

### 2.1. Participants

Twenty-one Texas State University undergraduates participated for course credit (12 females, 9 males, mean age = 20 ± 3 years, age range = 18–31). Note that the data for these participants were combined from data collected as part of two separate experiments devoted to exploring the EEG correlates of an unrelated visual categorization task (see Section 2.2: Resting State EEG Procedure, below); thus, the final sample size was conveniently determined by factors related to this categorization task and not the resting state task, according to the focus of this paper. All participants gave written informed consent in accordance with the Declaration of Helsinki. The Institutional Review Board at Texas State University approved this study.

### 2.2. Resting State EEG Procedure

After consent, participants underwent the setup for EEG recording. Participants completed several questionnaires during the setup that inquired about demographic and health information, quality and quantity of sleep, current sleepiness and attentional states, past and present emotion and mood states, and task-related mental workload. The questionnaire results are not reported here because they are not relevant to the topic of this paper.

After the EEG setup was finished, eight minutes of resting state EEG recording was obtained from each participant while they sat quietly on a comfortable padded chair in a darkened room with their eyes open or closed in interleaved 1 min intervals. A total of 4 min of EEG recordings were obtained for each resting state condition; eyes open/closed order was balanced across participants. Participants were directed to remain relaxed, alert, and awake during the EEG recording. After completing the resting state EEG recording, participants performed a visual categorization task, the results of which are not relevant to the topic of this paper and are not reported here.

### 2.3. EEG Recording and Preprocessing

**Figure 2:**
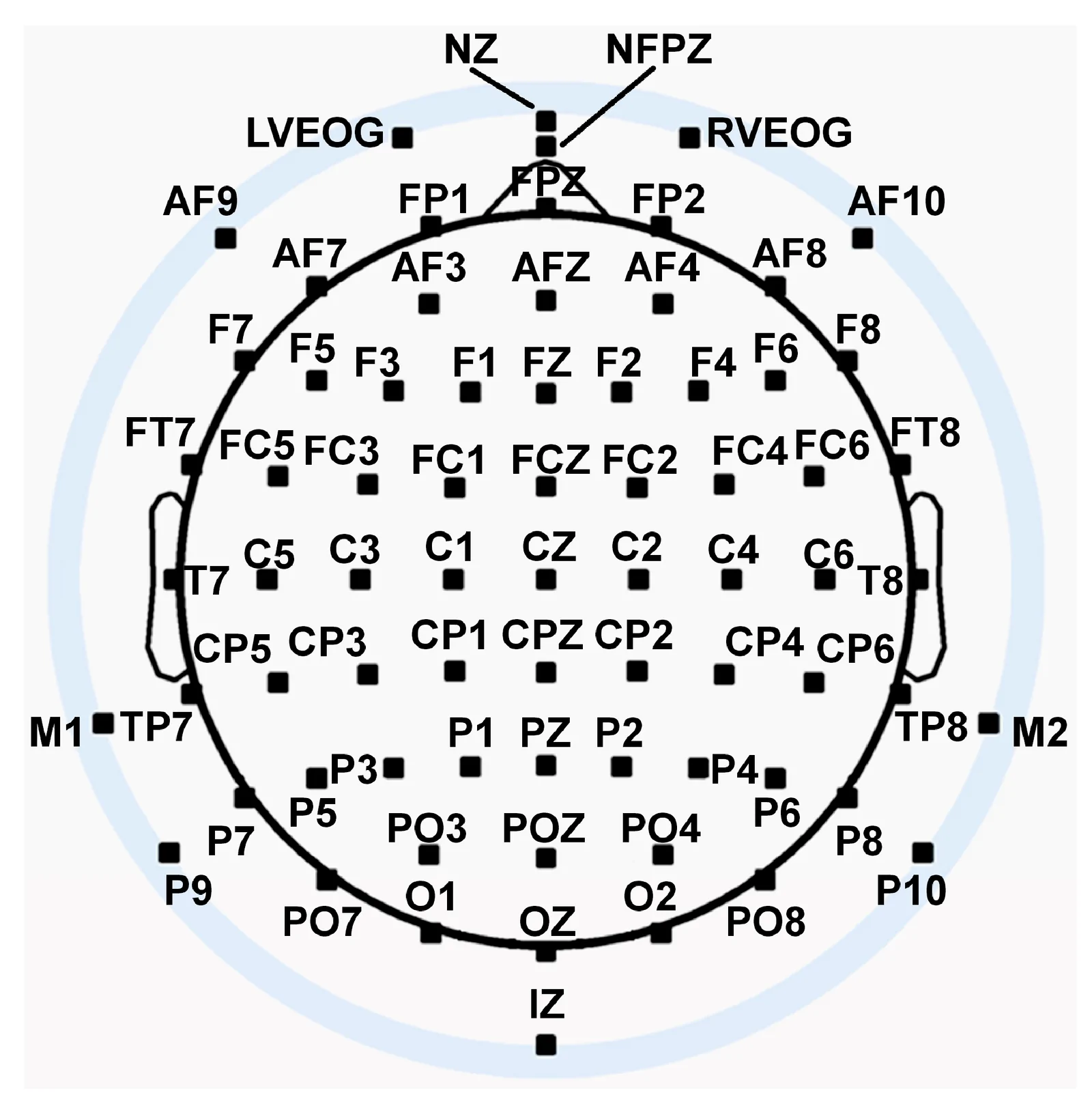
The scalp locations of EEG recording electrodes according to the extended international 10–20 system of nomenclature for EEG electrode placement. Electrodes displayed outside of the head radius were at locations below the equator (FPZ-T7-T8-OZ plane) of the assumed spherical head model. Electrodes LVEOG and HEOG were located below the eyes at approximately the same longitude as electrodes M1 and M2. Figure adapted from [24] with permission of the authors.

During the resting state, seventy-two EEG channels (Figure 2) were recorded from each participant’s scalp via a Biosemi Active II EEG system (Biosemi B.V., Amsterdam, The Netherlands) in 24-bit DC mode. The initial sampling rate was 2048 Hz, which was then downsampled online to 256 Hz; EEG signals were recorded relative to a common mode sense reference electrode located between sites PO3 and POZ. The EEG data were preprocessed using standard procedures [23,24,30], which included the removal of data contaminated by ocular, muscle, and signal artifacts. Bad EEG channels were replaced using spherical spline interpolation (m = 5; 50-term expansion) [31,32] applied to the other channels; the mean number of interpolated channels was 2.0 ± 2.0 (less than 3% of all channels). After pre-processing, the EEG data were then transformed into Laplacian-based current source densities (CSD: μV/m^2^ units) to diminish spatial mixing effects of volume conduction on the integration, complexity, and network measures [24,33,34] (see also Section 2.9.1, below). The Laplacian transformation was implemented using the CSD Toolbox for the MATLAB R2024b computing software (The MathWorks, Inc., Natick, MA, USA) [35,36,37].

Laplacian-transformed EEG data were bandpass filtered within the general alpha band range (7–13 Hz). However, initial inspection of fast Fourier transform (FFT)-based EEG spectral power density (see Section 2.5: Computation of EEG Power Spectral Density section, below) applied to the unfiltered EEG data revealed high variability in the alpha band frequency edges across participants and conditions. This observation is consistent with previous findings that alpha band characteristics may vary as a function of age, cognitive and neurological state, and task demands [38]. Thus, the alpha band was defined individually for each participant and condition as the frequency range within which EEG power deviated from a 1/f power law within the general alpha band frequency range. This approach identified the portion of the alpha range that predominantly reflects oscillatory network activity with a characteristic time scale rather than fractal (or scale-free) dynamics, as described in [39]. (However, unlike the latter study, the oscillatory and fractal alpha range components were not separated from one another for subsequent data analysis). The individual alpha band definitions were then used to define edge frequencies of a bandpass filter (513 point zero-phase shift finite impulse response filter; 1.65 Hz transition bands) for each participant and condition. On average, eyes-open condition edge frequencies ranged from 8.00 ± 0.95 Hz to 12.86 Hz ± 0.66 Hz, whereas eyes-closed condition edge frequencies ranged from 7.76 ± 0.70 to 12.62 ± 0.67. A signal following method [40] was used to avoid edge effects during bandpass filtering. Here, bandpass filtering was initially applied to 2 s EEG epochs with 75% overlap, before truncating the data to 1 s epochs with 50% overlap; data truncation was performed to reduce the computation time of integration, complexity, and network measures to a tractable level. On average, 321 ± 28 eyes-closed and 287 ± 84 eyes-open EEG data epochs were included in the final data analysis (after removal of epochs due to artifacts); the difference in trial number between resting state conditions was not statistically significant, *t*(20) = 1.51, *p* < 0.15.

Finally, all EEG signals were mean centered for each trial. Preprocessing analysis was also implemented using the EEGLAB toolbox [41] for the MATLAB R2024b computing software environment.

### 2.4. Dipole Modeling and Simulation of Resting State EEG Data

The goal of dipole modeling in this study was to understand how alpha range resting state neuroelectric source-level EEG properties (power, integration, complexity, modularity, and associated network properties) and corresponding scalp-level EEG properties varied as cortical EEG dipole sources changed their spatiotemporal dependencies among each other from fully random to fully dependent (or ordered). These dipoles approximated posterior cortical EEG sources known to be predominantly active during EEG resting tasks [42] and detectable by scalp EEG recordings [43]. The strategy here was not to provide an exact cortical model of the EEG resting state, but to emulate general coherent parieto-occipital alpha-range cortical activation in a manner that would roughly reproduce the large-scale patterns of resting state EEG signals observed at the scalp. This strategy is reasonable given that any measured scalp electromagnetic distribution may be described by an infinite number of possible source distributions [44].

#### 2.4.1. Head Volume Conductor and Dipole Location Model

**Figure 3:**
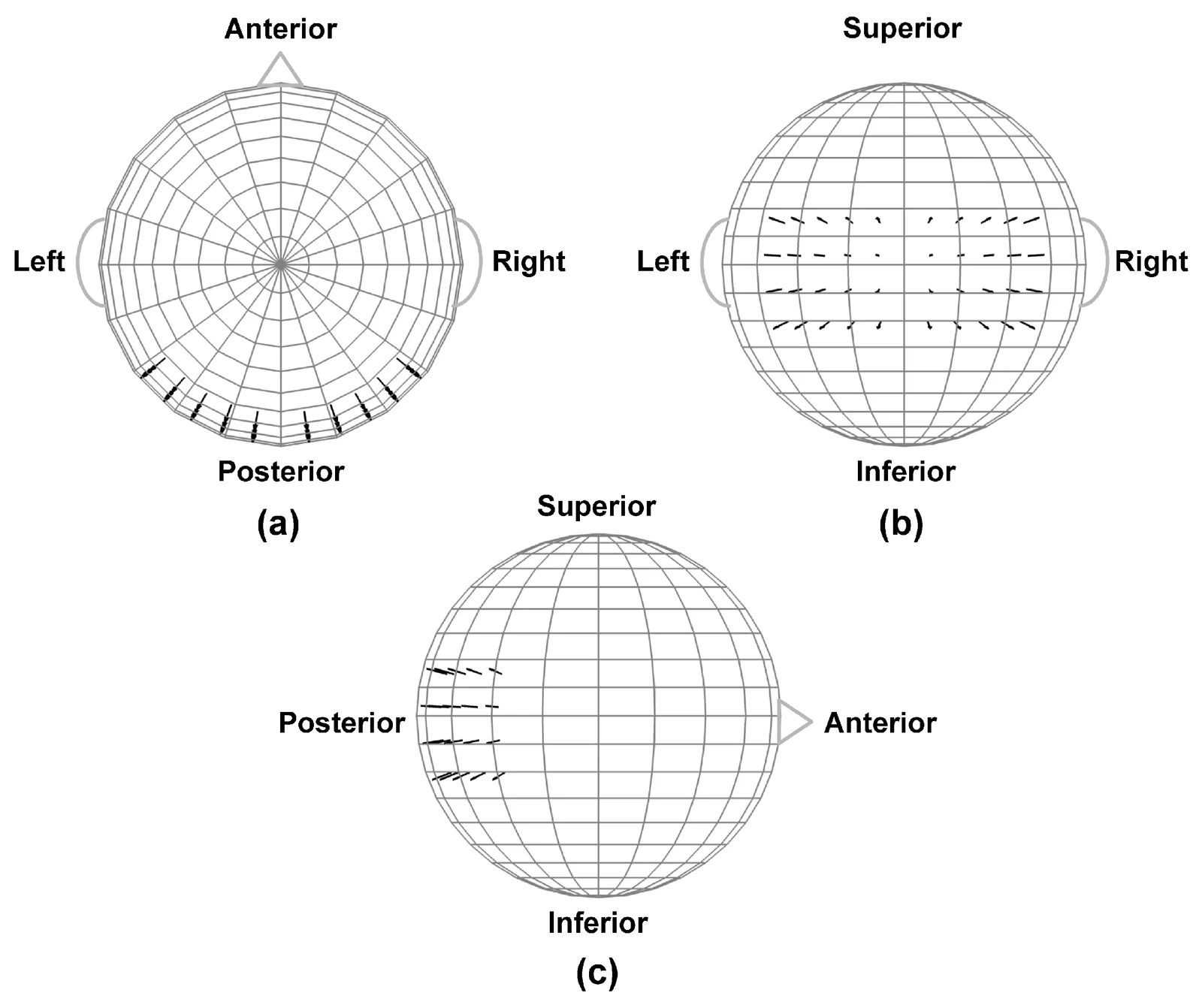
The outer layer of the 4-shell spherical head volume-conduction forward model. (**a**) Top head view; (**b**) Rear head view; (**c**) Side head view. Black arrows indicate the locations and orientations of the occipital dipole sources relative to the outer layer. All dipoles were placed in a radial orientation at superficial (2 mm subdural) cortical locations. The remainder of the model cortex surface was occupied by 148 equally spaced background activity dipoles. Figure adapted from [23,24] with permission of the authors.

The neuroelectric sources were modeled as radially directed current dipoles positioned within a concentric 4-shell spherical head-forward volume-conduction model [45,46,47] implemented via in-house MATLAB scripts. In this model, the cortex surface was placed at a 76 mm radius (*σ_cortex_* = 0.33 S/m), which was covered by a 2 mm cerebrospinal fluid layer (*σ_CSF_* = 1 S/m). The model’s outer shell had an 85 mm radius, with a scalp thickness = 3 mm (*σ_scalp_* = 0.33 S/m) and bone thickness = 4 mm (*σ_bone_* = 0.0042 S/m). Shell/layer thickness and conductivity values were taken from [45,46]. Two clusters of 24 radially oriented dipole generators (48 dipoles total) were created at posterior, roughly occipital locations (Figure 3) just below the cortical surface (2 mm subdural, following [47]). One cluster was placed in each hemisphere with the anterior–posterior location of both clusters centered on the equator (FPZ-T7-T8-OZ) of the spherical head model. The remainder of the model cortex surface was filled with 148 equally spaced dipoles to simulate background cortical processes (termed here background dipoles). Model scalp electrode sites were located on the model scalp surface at the same extended 10–20 system locations used for the EEG recordings (see Figure 2), with simulated scalp potentials computed at these locations via forward volume conduction transformation. This specific head volume conductor and dipole location model has previously been used to study EEG integration and complexity [23,24].

#### 2.4.2. Simulation of Functional Modularity

**Figure 4:**
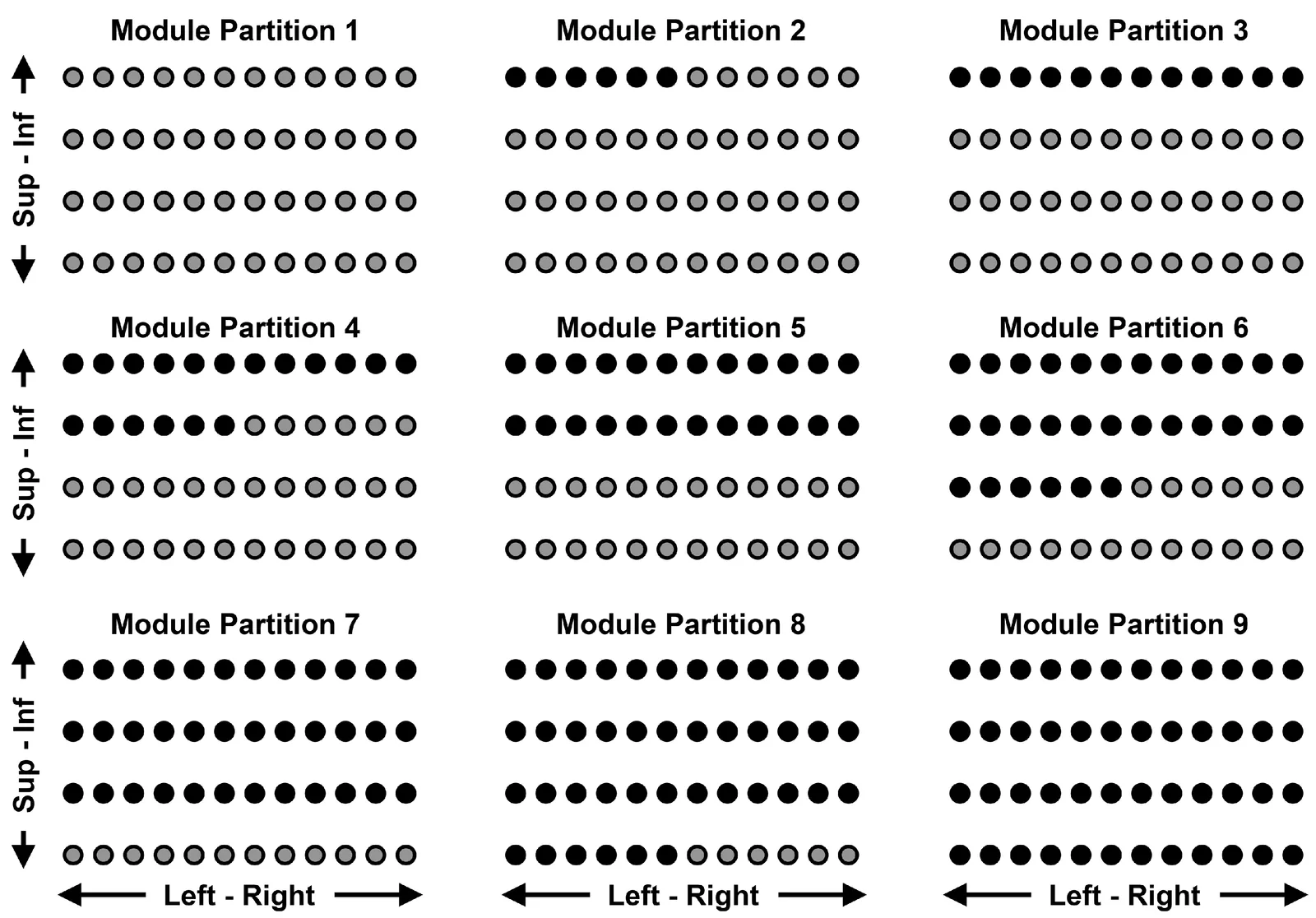
Phase 1 dipole model spatiotemporal dependency manipulation (Dipole Model Manipulation Phase 1). Occipital dipoles are shown in a two-dimensional array as viewed from the back of the simulated brain. Dipoles spanned superior to inferior (Sup–Inf) and left to right (Left–Right) locations across the simulated cortical surface. Grey shaded circles indicate aperiodic time courses; solid dark circles indicate periodic time courses. See text for additional details.

Simulation of how source- and scalp-level EEG properties varied with changes in the modularity of the forty-eight occipital dipole source moments was achieved by manipulating the spatiotemporal dependencies in an organized manner to create eighteen functional module partitions of the dipoles. This manipulation was implemented in two phases. In the first manipulation phase (Dipole Model Manipulation Phase 1, Figure 4), all occipital dipole source moments were initially created with aperiodic temporally randomized time courses and amplitudes (Module Partition 1); this partition simulated spatiotemporally independent random signals. Next, the temporally random time courses of neighboring clusters of six dipole sources were replaced with periodic time courses modeled as sine waves characterized by randomized amplitude, frequency, and phase variations that simulated independent, unsynchronized alpha-range EEG oscillations (see below). Initially, only one dipole cluster was transformed in this manner (Module Partition 2); then, the number of clusters increased across seven additional steps (Partitions 3–9; see Figure 4) until all forty-eight occipital dipole source moments were periodic with random amplitudes, frequencies, and phases (Partition 9; see Figure 4). This manipulation phase simulated a transition of the occipital EEG source signals from being relatively random and independent in time and space to periodic, temporally dependent signals that were relatively spatially independent.

**Figure 5:**
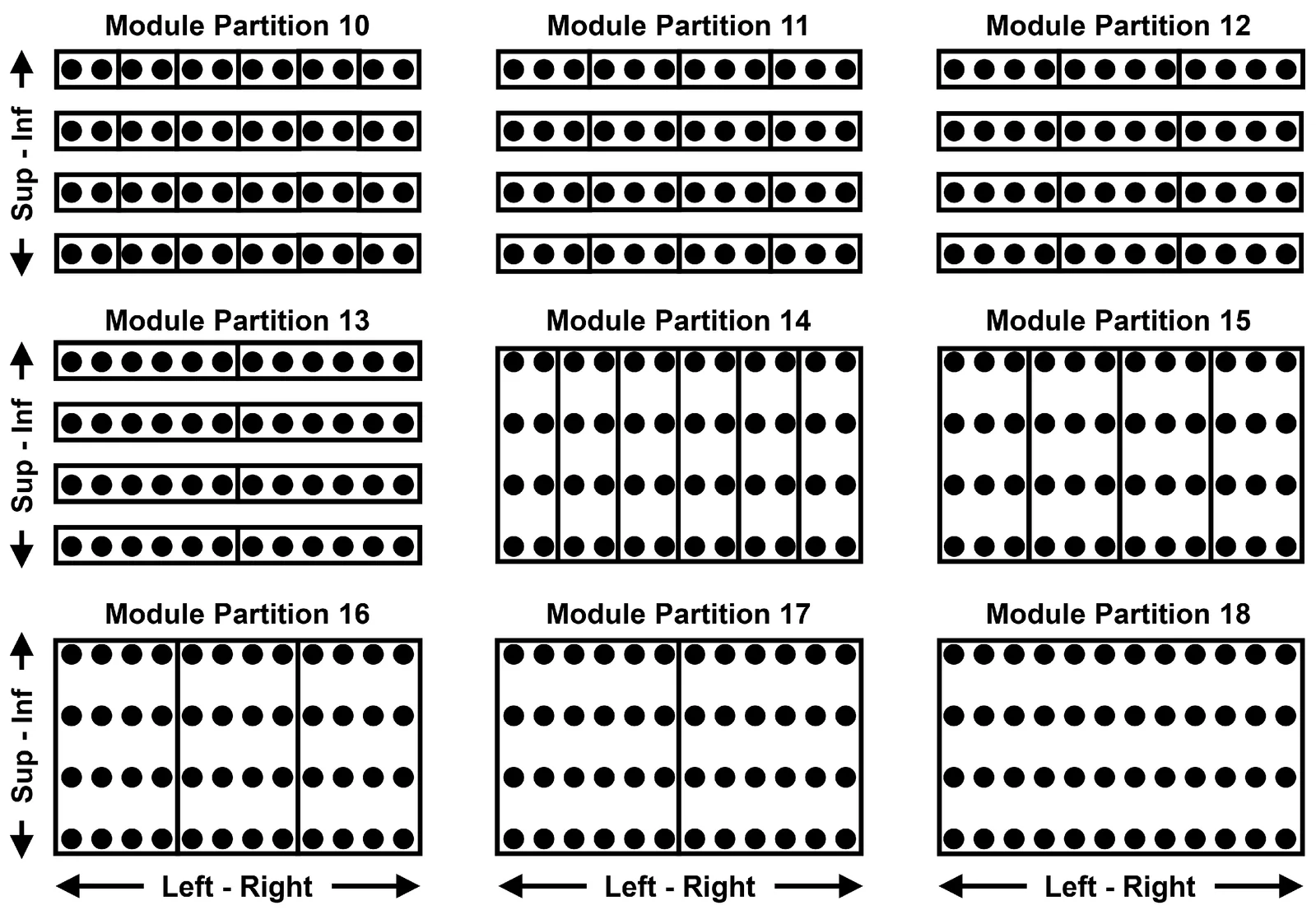
Phase 2 dipole model spatiotemporal dependency manipulation (Dipole Model Manipulation Phase 2). Occipital dipoles are shown in a two-dimensional array as viewed from the back of the simulated brain. Dipoles spanned superior to inferior (Sup–Inf) and left to right (Left–Right) locations across the simulated cortical surface. Solid dark circles indicate periodic time courses; boxes indicate module clusters. See text for additional details.

In the second manipulation phase (Dipole Model Manipulation Phase 2, Figure 5), the spatiotemporal dependencies among the periodic occipital dipole source moments were manipulated further by replacing the time courses of neighboring clusters of dipole source moments with sine waves of identical amplitude and frequency but with uniformly distributed random phase shifts (±π). Thus, each cluster of dipole moments realized a high-level of synchronization and strong spatiotemporal dependence among the signals necessary to form a functional module. All modules in each Phase 2 partition were the same size, with the number and size of the modules chosen to evenly partition the forty- eight dipole sources, e.g., Partition 10 consisted of twenty-four two-dipole modules, Partition 11 consisted of sixteen three-dipole modules, etc. (see Figure 5). Partition 18 was formed by one module consisting of forty-eight dipoles, each with identical amplitude and frequency but with phases that differed randomly by a small amount (±0.35 radians). This final partition simulated near-perfect spatiotemporal dependency among all the posterior dipole sources. EEG time courses and scalp distributions were computed for each source partition via forward volume-conduction transformation to the scalp. Then, the integration *I*(*X*), interaction complexity *C_I_*(*X*), and network parameters of both the dipole sources and the scalp signals were computed.

Each dipole simulation consisted of 108 1 s trials (256 Hz sampling rate) created via in-house MATLAB scripts. During each simulated trial, the magnitude of each dipole source moment sinusoidally oscillated over time, as characterized by amplitude, frequency, and phase variations chosen to simulate alpha range EEG states. Periodic occipital dipole sources were created from sinusoids with uniformly random frequencies (7–13 Hz) and starting phases (0–2π radians) that were constant across timepoints. Dipole sources with temporally randomized time courses were created from sinusoids with frequencies and phases that were randomly selected at each point in time according to a uniform distribution (frequency: 7–13 Hz; phases: 0–2π radians). Amplitude fluctuations of the visual dipole sources were modeled by multiplying each simulated dipole moment time course by a Gaussian window (*σ_t_* = 50 ms) that was circularly shifted randomly along the time dimension of each trial. The circular shifting of the window was randomized according to a uniform distribution for each dipole time course and trial separately. Occipital dipoles had a maximum dipole source moment of 20 μA-cm. A small amount of uniformly distributed noise (±1% of the maximum dipole source moment magnitude) was added to each timepoint of the dipole moment time course. Finally, the remaining 148 background dipole sources were created from sinusoids with uniformly random frequencies (7–13 Hz) and starting phases (0–2π radians) that were constant across timepoints and constant moments that were 50% of the strength of the extrastriate dipole sources.

Scalp potential topographies and time courses for each individual dipole generator were computed via forward volume-conduction transformation to the scalp. The final simulated EEG scalp record was constructed from the sum of the individual dipole topographies at each timepoint, following the linearity of the volume-conduction transformation [47]. The simulated scalp EEG signals were then Laplacian-transformed, filtered in the alpha-band range, and mean-centered for each trial and electrode (see Section 2.3: EEG Recording and Preprocessing, above). Then, the EEG properties of the sources and the scalp signals were computed.

### 2.5. Computation of EEG Power Spectral Density (PSD)

Empirical and simulated resting EEG power spectral density (PSD) was computed on unfiltered data epochs via fast Fourier transformation (FFT) after tapering the EEG signals using a 1 s Hamming window [23,24]. For each participant and resting state condition, PSD was computed in its original units (μV^2^/Hz) and then converted into decibels (dB). Mean PSD values were computed over each participant’s individually defined alpha-band ranges for statistical testing and the general alpha range (7–13 Hz) for visualization purposes. Mean PSD was computed at bilateral posterior scalp sites PO7 and PO8, which were chosen because they demonstrated maximal PSD responses in the alpha-band frequency range (see Section 3.1.1: Observed EEG Power Spectral Density (PSD), below).

### 2.6. Computation of EEG Signal Regularity

Estimation of empirical and simulated resting EEG signal regularity was computed using the run test for randomness (*RTR*) [48], which tests the null hypothesis (*RTR* = 0) that the values in a time series are sequenced in a random order versus the alternative hypothesis (*RTR* = 1) that they are non-random or regular. If the test returns *RTR* = 1, then the null hypothesis is rejected at the two-tailed *p* < 0.05 significance level, whereas if the test returns *RTR* = 0, then the test fails to reject the null hypothesis at the two-tailed *p* < 0.05 significance level. The *RTR* test was implemented via the MATLAB runstest.m function using default values. The test was applied to each individual dipole source or scalp electrode signal on a given trial, with test results averaged across electrodes and trials for each participant and condition. Moreover, the *RTR* test was applied to both filtered and unfiltered data scalp-level empirical and simulated data due to the likelihood that the smoothing operation of a band-pass filter could artificially impose regularity on random signals.

### 2.7. Computation of EEG Functional Integration and Topological Complexity

Here, each data trial consisted of *N_s_* EEG signals unfolding simultaneously over *N_t_* timepoints. Thus, the data at each timepoint were considered as a set of vectors of individual EEG signals *X_j_* = [*x*_1_ *x*_2_ … *x_i_* … *x_Ns_*] that collectively formed a multidimensional EEG data space *X* = {*X*_1_ *X*_2_ … *X_j_* … *X_Np_*}, where the dimension of the space *d* = *N_s_* and the number of data points in the space *N_p_* = *N_t_*. Functional EEG integration and topological complexity were then computed via two information-theoretic measures that quantify statistical dependence among multiple variables, the Tononi–Edelman–Sporns integration *I*(*X*) and interaction complexity *C_I_*(*X*) [4,6], expressed as follows:

(1) $$I(X)=\sum_{i=1}^{d}H(x_{i})-H(X),$$

(2) $$C_{I}\left(X\right)=H\left(X\right)-\sum_{i=1}^{d}H\left(x_{i}|X-x_{i}\right).$$

The *I*(*X*) and *C_I_*(*X*) measures are formed from the combination of different entropies *H* that quantify the uncertainty, and thus informativeness, of the EEG signals, expressed as follows:

(3) $$H\left(X\right)=-E\left[\log\left(f\left(X\right)\right)\right]=-\int f\left(X\right)log(f\left(X\right)dX$$

where *f*(*X*) is the multivariate probability density function at each point *X_j_* in the EEG signal space. In Equations (1) and (2), *H*(*x_i_*) is the entropy of an individual EEG channel *x_i_*, *H*(*X*) is the joint entropy of the coincident patterns *X* across all EEG signals, and

(4) $$H\left(x_{i}|X-x_{i}\right)=H\left(X\right)-H\left(X-x_{i}\right)$$

is the conditional entropy of a single EEG signal *x_i_*, given the state of the other EEG signals *X* − *x_i_*. *I*(*X*) indexes the overall deviation from statistical independence of the individual elements in a system, whereas *C_I_*(*X*) is a statistical measure of a system’s information content that results from the interactions among the system’s elements. *I*(*X*) = 0 when all EEG signals are statistically independent; otherwise, it is maximal when the EEG signals are fully dependent. In contrast, *C_I_*(*X*) is minimal for fully independent or dependent channels and is maximal when the channels are neither completely independent nor completely dependent; see Figure 1.

In practical applications involving high-dimensional data with unknown and possibly nonstationary probability distributions, the exact computation of Equation (3) is intractable. To circumvent this difficulty, a nonparametric geometric k-nearest neighbors (G-KNN) estimator [49] of the entropy of a data space was used to the calculate univariate and multivariate versions of *H* utilized in Equations (1) and (2). This approach has been previously used to compute the integration and complexity of resting state EEG data [23]. The G-KNN entropy estimator accurately models irregularities in the local geometry of a data space by using singular value decomposition (SVD) to compute volumetric elements of the neighborhood of each data point [49], as follows:

(5) $$H_{G-KNN}(X)=\log(N_{p})+\log(\pi^{\frac{d}{2}}/\Gamma (1+d/2))-\frac{1}{N_{p}}\sum_{j=1}^{N_{p}}log(k(X_{j}))\;+\frac{d}{N_{p}}\sum_{j=1}^{N_{p}}log(\varepsilon (X_{j},k))+\frac{1}{N_{p}}\sum_{j=1}^{N_{p}}\sum_{l=1}^{d}log(\frac{\sigma_{j}^{l}}{\sigma_{j}^{1}})$$

where *k*(*X_j_*) is the number of neighborhood points within the volume of an ellipse centered on that point, *ε*(*X_j_*,*k*) are the Euclidean *k*-th nearest-neighbor distances, and *σ^l^_j_* are the singular values for each point *X_j_*. The specific procedure for computing *H_G-KNN_*(*X*) can be found in [49]. Note that in the case of *d* = 1, *log*(*σ^l^_j_/σ*^1^*_j_*) = *log*(*σ*^1^*_j_/σ*^1^*_j_*) = 0, Equation (5) reduces to a variant of the Kraskov–Stögbauer–Grassberger (KSG) KNN entropy estimator [50,51], as follows:

(6) $$H_{KSG}(x_{i})=\log(N_{p})+\log(\pi^{\frac{d}{2}}/\Gamma (1+d/2))-\frac{1}{N_{p}}\sum_{j=1}^{N_{p}}\log(k(x_{ij}))+\;\frac{d}{N_{p}}\sum_{j=1}^{N_{p}}\log(\varepsilon (x_{ij},k)).$$

For the computation of *I*(*X*) and *C_I_*(*X*) in the present study (following [23]), Equation (5) was used to compute joint entropy *H*(*X*) and conditional entropy *H*(*x_i_|X − x_i_*) (where the second term of Equation (4) is computed by redefining *X′* = *X* − *x_i_*), and Equation (6) was used to compute the individual channel entropies *H*(*x_i_*).

Integration *I*(*X*) and interaction complexity *C_I_*(*X*) were computed for empirical and simulated EEG signals by estimating *H*(*X_i_*), *H*(*X*), and *H*(*x_i_|X − x_i_*) from data pooled across each individual EEG data epoch. This is equivalent to treating each set of EEG signals as a jointly distributed space with the number of dimensions equal to the number of signals and each timepoint representing an individual observation drawn from the joint distribution. *I*(*X*) and *C_I_*(*X*) were computed for each preprocessed EEG trial before averaging EEG integration and complexity across trials to produce a final estimate. EEG integration and complexity were computed for each participant and resting state condition (eyes open, eyes closed). All *I*(*X*) and *C_I_*(*X*) values were computed in units of bits.

The value of the nearest-neighbor free parameter *k* was chosen to be three times the number of simulated EEG sources (*k_source_* = 3 × 48 = 144) and scalp EEG channels (*k_scalp_* = 3 × 72 = 216). The value of *k* should be small enough to provide a local estimate of the geometry of the variable space but large enough to allow a proper estimate of the shape of the local geometry [49]. Additionally, consistent estimation of the multivariate probability density used to compute the entropy of a data space requires *k* to grow with the number of data samples [52,53], which for the present empirical and simulated data was 256 samples for each 1 s epoch sampled at 256 Hz. Moreover, the value of *k* for the G-KNN estimator must greater than or equal to the dimension of the joint variable space of the data under analysis [49]. The choice of *k_source_* = 144 for the simulated EEG sources and *k_scalp_* = 216 for the scalp EEG channels (empirical and simulated) was a compromise that balanced these criteria. Although the use of relatively large *k* values may have affected the accuracy of entropy estimation, this was likely mitigated by the fact that the variance of G-KNN entropy estimates is reduced when using larger values of *k* [52,54]. Furthermore, although the KSG estimator (Equation (6)) only acts on a 1-dimensional variable space when computing *H*(*x_i_*), and the *G-KNN* estimator (Equation (5)) operates on a space with a number of dimensions reduced by 1 relative to the full joint variable space when computing *H*(*x_i_|X − x_i_*), the same values of *k_source_* and *k_scalp_* was used for all entropy estimates in order to avoid potential scaling differences that might have arisen from the use of different values of *k* across the different estimations (following [23]).

### 2.8. Estimation of Brain Network Modular Organization

#### 2.8.1. Functional Connectivity

Analysis of brain modularity began with computation of the functional connectivity matrix. For each EEG dipole simulation, functional connectivity matrices were separately created at the source level and at the scalp level. For the empirical EEG data, functional connectivity matrices were created only at the scalp level.

##### Simulated Dipole Sources

For the simulated EEG dipole sources, each dipole source was considered a network node to then construct 48 × 48 undirected connectivity matrices using Pearson correlation coefficients between the pairwise time courses of each dipole source, with a separate matrix created for each trial of each dipole simulation. Following standard recommendations [55], autocorrelation values and negative weights were set to zero. However, contrary to typical network analyses, thresholding and binarization were not applied to the connectivity matrix, which remained weighted for all analyses. This choice was made for two reasons. First, this choice simplified the overall analysis because several levels of thresholding and binarization are typically required to fully capture the connectivity of a network across local and global scales [27]. Second, using a weighted adjacency matrix should capture the continuous variation of connectivity among brain elements in a manner that parallels the continuous variation of statistical dependence among brain signals captured by the information-theoretic functional integration and topological complexity measures used in this study (see Section 2.7: Computation of EEG Functional Integration and Topological Complexity, above). Thus, this approach should provide a clearer comparison between the brain network measures and the measures of functional integration and topological complexity used in this study. Separate connectivity matrices were created for each trial of each dipole simulation.

##### Simulated and Empirical Scalp-Level Signals

At the scalp level, each EEG sensor was considered a network node to construct 72 × 72 undirected connectivity matrices from the simulated and empirical scalp EEG signals using a modified version of phase locking value (*PLV*), a widely used connectivity metric that indexes how the phases of two signals are coupled to each other. The modified version used here involved computing the normalized imaginary component of the standard *PLV* measure and is called corrected imaginary PLV (*ciPLV*) [56], expressed as follows:

(7) $$ci{PLV}_{ij}=\left|\frac{\frac{1}{T}I\left\{\sum_{t=1}^{T}e^{i(\theta_{i}-\theta_{j})}\right\}}{\sqrt{1-{\left(\frac{1}{T}R\left\{\sum_{1}^{T}e^{i(\theta_{i}-\theta_{j})}\right\}\right)}^{2}}}\right|$$

where *T* = number of timepoints, *θ_i_* = phase of signal *i*, *θ_j_* = phase of signal *j*, and *ℑ*{*x*} and *ℜ*{*x*} stand for the imaginary and real parts of *x*, respectively. Signal phases were extracted via wavelet-based time–frequency transformation [40] using normalized complex Morlet wavelets with center frequencies ranging from 7 to 13 Hz (7 linearly spaced wavelets over this range) and free scaling parameter *α* = *π*. The latter parameter choice ensured that the center portion of each wavelet spanned one cycle for each frequency; this allowed for more precise estimation of the temporal variation of each signal phase. The *ciPLV* connectivity measure takes on values from 0 to 1, where 0 indicates an absence of connectivity and 1 indicates perfect connectivity. This measure was used because it mitigates spurious correlations between electrodes due to volume conduction of EEG source signals through the head by ignoring zero-lag correlations [56]. Moreover, this measure was chosen rather than the weighted phase lag index (*wPLI*) [57] that is often used in the brain connectivity literature because PLI-based measures comparatively show lower test-retest reliability [58,59]. Similar to the source-level connectivity matrix, autocorrelation values were set to zero for all scalp-level connectivity matrices. Separate connectivity matrices were created for each trial of each dipole simulation and each trial, condition, and participant for the empirical EEG data.

#### 2.8.2. Detection and Analysis of Brain Network Modular Structure

The nodes of a brain network can be subdivided into different partitions based on specific patterns of connectivity among the nodes. Network communities or modules are subdivisions of densely connected network nodes that “are more strongly connected with each other than with other parts of the network” [27] (p. 303). In the present study, brain network modular structure was detected via nested iterative modularity maximization using the Louvain community detection algorithm [60] as implemented by the community_louvain.m function of the Brain Connectivity Toolbox (*BCT*) [55]. Modularity *Q* is a statistic that quantifies the “strength” of a network partition, i.e., the degree to which a network can be partitioned into non-overlapping groups of nodes such that the numbers of within-group edges are maximized, and the numbers of between-group edges are minimized [55]. Modularity *Q* was computed as follows [61,62,63]:

(8) $$Q=\frac{1}{W}\sum_{ij}\left(w_{ij}-{\gamma g}_{ij}^{w})\delta (m_{i},m_{j}\right)$$

where $W=\sum_{ij}w_{ij}$ is the sum of all weights in the network, $g_{ij}^{w}=s_{i}s_{j}/W$ the connectivity weights linking nodes in the same community that is expected by chance, *s_i_* and *s_j_* are the strengths of nodes *i* and *j*, *m_i_* is the module containing node *i*, and $\delta \left(m_{i},m_{j}\right)=1$ if *m_i_* = *m_j_* and 0 otherwise. The parameter *γ* sets the resolution of community detection, with *γ* > 1 biasing towards the detection of smaller communities and *γ* < 1 biasing towards the detection of larger communities (*γ* = 1 reflects the original definition of *Q* [64]). The *Q* statistic is associated with a modularity partition of the network, which is described by a community affiliation vector *M* whose entries are numbers indicating the specific module to which a node belongs. Thus, *M* contains information about the specific partitioning of network nodes into modules as well as the total number *N_M_* of modules in a partition.

The Louvain community detection algorithm works by first optimizing *Q* locally on all nodes to detect small modules, merging these small modules into single nodes, and then repeating these steps until *Q* is maximized and no further increases in *Q* can be achieved [27,60]. However, such modularity maximization suffers from two weaknesses that must be sufficiently addressed to achieve confident results. First, it has a resolution limit that arises from a bias against the detection of smaller communities [27]. Second, it suffers from a degeneracy problem [27] arising from the fact that in general, a network affords many possible partitions, each with a modularity *Q* that differs slightly from the partition that yields the maximum value of *Q_max_* for a network [65]. These resolution and degeneracy concerns were addressed in the present study by implementing community detection as a nested 3-level structure of iterations. The iterations inherent to the Louvain algorithm itself established the 1st level of iteration, whereas the 2nd level of iteration was implemented by sequentially repeating the Louvain algorithm such that the community affiliation *M* output by one instance of the algorithm became the initial community affiliation input into the next instance of the algorithm. Second-level iteration was performed until the change in modularity was less than 1 × 10^−5^. The starting community affiliation *M*_0_ set at the beginning of a 2nd-level iteration sequence was set such that the number of partitions/modules was initially equal to the total number of network nodes (48 nodes for the source-level networks and 72 nodes for the scalp-level networks), with the initial label of each partition randomly selected without replacement from the range 1 to the number of network nodes. The 1st and 2nd levels of iteration were then nested within a 3rd level of iteration in which the *γ* parameter input into each 2nd-level iteration of the Louvain algorithm was iteratively adjusted to an optimal value for a given modularity partition. This optimization was achieved using a maximum likelihood-based method [66], as follows:

(9) $$\gamma =\frac{\omega_{in}-\omega_{out}}{log(\omega_{in})-log(\omega_{out})}\;\omega_{in}=\frac{2m_{in}}{\sum_{r}\kappa_{r}^{2}/2m}\;\omega_{out}=\frac{2m-2m_{in}}{2m-\sum_{r}\kappa_{r}^{2}/2m}$$

where *m* is the total number of edges, *m_in_* is the total number of in-module edges, and *κ_r_* is the sum of the degrees of the nodes in module *r*. Computation of network density for Equation (9) was computed using the BCT density_und.m function. For each run of 3rd-level iterations, the initial starting value for *γ* was 1, with the iterations performed until the change in *γ* was less than 1 × 10^−2^. It should be noted that the maximum likelihood method for estimating *γ* has certain limitations, which are discussed in Section 4.6: Study Limitations, below.

The modularity degeneracy problem was further addressed in the present study through consensus-based clustering [27]. This method assesses how consistently network nodes are assigned to the same modules across different partitions. This consistency is then used to create a consensus partition that is a representative summary of the modular structure across a set of individual partitions that represents the community structure of a network in a way that is robust to the degeneracy problem. Here, module partition consensus was achieved by performing 20 different runs of the nested 3-level iterative modularity maximization described above for each brain network. Each run then produced slightly different near-optimal *Q*, *M*, and *γ* values. A consensus partition was then created from the 20 *M* vectors using the agreement.m and consensus_und.m functions of the BCT. This consensus partition was then used to select a final partition *M_F_* used for subsequent analysis from the set of 20 modularity-maximized partitions that were most similar to the consensus partition. This was achieved by selecting *M_F_* as the individual partition with the smallest mean squared error between the consensus partition and each partition across the 20 modularity-maximized partitions. It should be noted that modularity maximization more generally has additional limitations, which are discussed in Section 4.6: Study Limitations, below.

Five metrics of network community structure were calculated from the representative modular partition *M_F_*, modularity statistic *Q*, total number of modules per partition *N_M_*, mean module size *M_Size_*, mean participation coefficient *PC*, and mean participation index *PI*. The mean participation coefficient *PC* for a network is a summary metric that quantifies how the connections of the network’s nodes are distributed across different modules [67]. Initially, the participation coefficient was computed for each individual node as follows:

(10) $${PC}_{i}=1-\sum_{m=1}^{N_{M}}{\left(\frac{k_{im}^{W}}{k_{i}^{W}}\right)}^{2},$$

where *Ns* is the number of EEG signals, *N_M_* is the number of modules in the network, *k_i_* is the weighted degree of node *i*, and *k_im_* is the sum of the weights between node *i* and the other nodes in module *m*. The participation coefficient is derived from the community affiliation vector *M* and quantifies the distribution of a node’s links across modules; *PC* = 1 when a node’s links are uniformly distributed across modules, *PC* = 0 when a node’s links are all contained within a single module. The participation coefficient was computed via the participation_coef.m function of the BCT. The summary *PC* value for a network was then computed by averaging *PC_i_* across the network’s nodes.

The participation index *PI* is a variant of the *PC* that quantifies how a node’s connections are distributed across different modules in a manner that controls for biases that might emerge when comparing across networks with different numbers and sizes of modules [68]. *PI* was computed as validity check on the more traditional *PC* metric, whereas the more widely used *PC* metric was computed to provide a clearer comparison of the present study with previous findings in the research literature. The participation index was computed by first calculating the probability *p* that a network node *i* belongs to module *m*, according to the following:

(11) $$p_{im}=\frac{k_{im}}{N_{im}},$$

where *N_im_* is the size of module *m* for node *i*. These probabilities were then normalized,

(12) $$\sum_{m=1}^{N_{M}}p_{im}=1,$$

and the participation index *PI_i_* for node *i* was computed as follows:

(13) $${PI}_{i}=1-\frac{N_{iM}}{\sqrt{N_{iM}-1}}\sigma (P_{i})$$

where **P**_i_ = (*p_i_*_1_, …, *p_im_*) and *N_im_* is the number of modules for node *i*. *PI_i_* = 1 when a node’s links are uniformly distributed across modules, whereas *PI_i_* = 0 when a node’s links are all contained within a single module. The participation coefficient was computed via an in-house modification of the participation_coef.m function of the BCT. The summary *PI* value for a network was then computed by averaging *PI_i_* across the network’s nodes.

For the dipole simulations, community detection and modularity maximization were performed for each epoch of each simulation at the source and scalp levels, with final reported values consisting of averages and standard deviations across epochs for each dipole simulation. For the empirical data, community detection and modularity maximization were performed for each epoch, condition, and participant, with final reported values consisting of averages and standard deviations across epochs and participants for each condition. Mean *Q*, *PC*, and *PI* values were converted to percentages of the original 0 to 1 range of these measures to more easily report small differences across conditions.

### 2.9. Statistical Analysis of Empirical EEG Measures

EEG signal measures underwent two stages of statistical testing. First, the EEG signal measures were assessed for the contribution of volume conduction-related spurious interdependencies via surrogate testing. Second, potential differences in EEG signal measures were assessed via non-parametric statistical testing.

#### 2.9.1. Surrogate Statistics

Scalp-level EEG properties (spectral power, integration, complexity, network measures) were initially assessed using a surrogate data technique to account for spurious dependencies present in scalp-level signals resulting from volume conduction [69,70,71]. Surrogate EEG data were created by superimposing EEG sources made statistically independent by randomly shifting their signal phases. The surrogate data and observed data were similar in terms of energetic and temporal characteristics. However, any EEG signal measures computed on the surrogate data should reflect volume conduction alone.

The method used here was a modification of that used by [23,24,72,73]. First, independent components cnalysis (ICA) [74] was used to decompose the empirical EEG data into quasi-independent signals. Then, each ICA component activation time course was randomly shifted to reduce the presence of any remaining statistical dependencies among the ICA components. Here, activation time courses within a trial were circularly shifted randomly within that trial. Finally, the ICA inverse mixing matrix was used to transform the shifted ICA activations back into EEG sensor space to yield the surrogate EEG signals.

This surrogate-creation procedure was performed on the original preprocessed resting state EEG signals. Integration, complexity, and network measures were then computed for each set of surrogate data. The mean and two-tailed 95% confidence intervals (*CIs*) of the surrogate integration and complexity were computed from 50 surrogate data sets created for each resting state condition and participant. Observed mean values with confidence intervals outside of the surrogate confidence intervals were accepted as significantly different from EEG property values arising from spuriously coincident volume-conducted EEG activity. Surrogate data testing was performed via custom MATLAB scripts, with ICA decompositions implemented using the extended infomax runica algorithm (with data whitening and default stopping weight change = 1 × 10^−7^) of the EEGLAB toolbox for MATLAB [41].

#### 2.9.2. Parametric Statistics

Reported data descriptive statistics include means, standard deviations (SDs), and 95% confidence intervals (95% CIs). Potential differences between EEG resting state conditions were statistically analyzed via non-parametric randomized permutation *t*-tests (5000 permutation) [75]. These tests were used when comparing between-condition differences for all EEG properties (spectral power, integration, complexity, network modularity plus associated measures). Nonparametric permutation Pearson correlations *R* were used to assess relationships between relevant pairs of between-condition differences. Uncorrected permutation tests were computed using the PERMUTOOLS MATLAB toolbox [76]. Then, all reported *p*-values were corrected for multiple comparisons using the Holm–Bonferroni procedure [77].

## 3. Results

### 3.1. Observed EEG Data

#### 3.1.1. Observed EEG Power Spectral Density (PSD)

**Figure 6:**
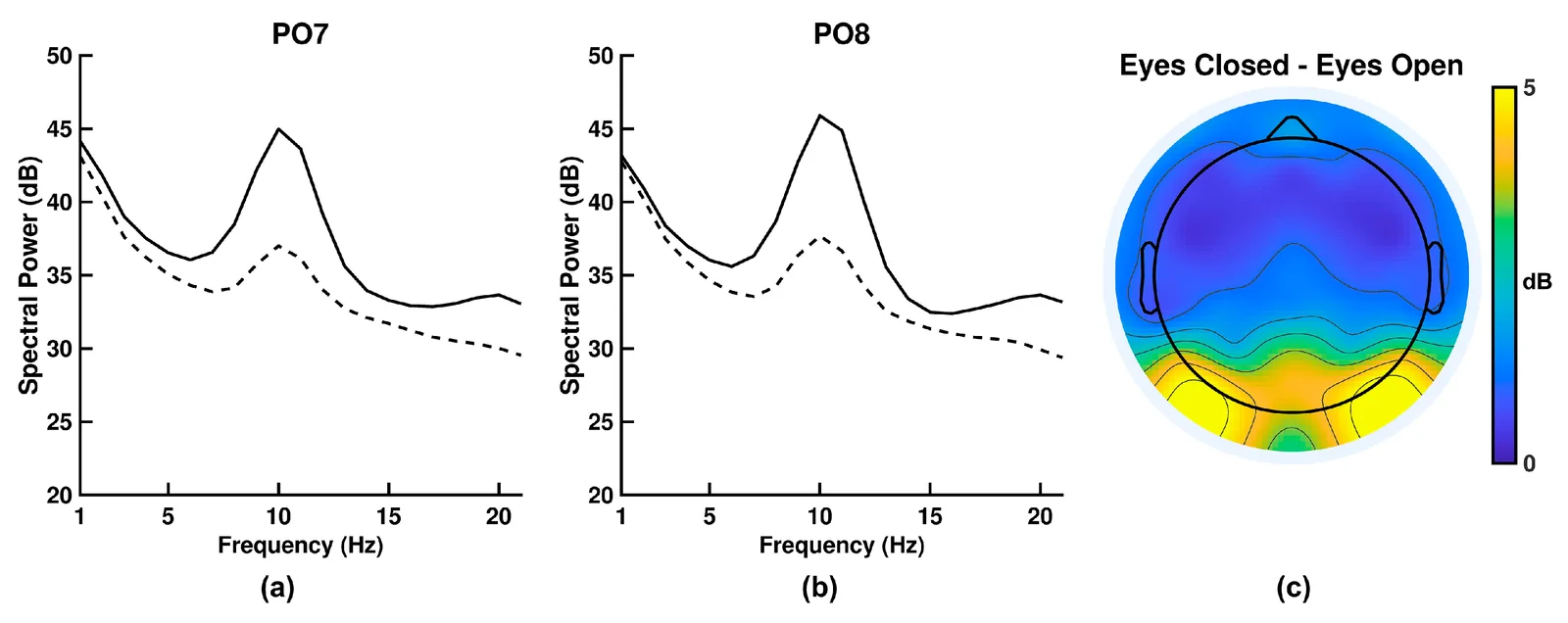
Resting state EEG spectral power density (*PSD*, in decibels) for eyes-closed (solid line) and eyes-open (dashed line) conditions. (**a**) PSD at site PO7; (**b**) PSD at site PO8; (**c**) Head map showing the scalp topography of the average difference between eyes-closed versus eyes-open alpha-range (7–13 Hz) *PSD*.

Plots of resting state EEG *PSD* are shown in Figure 6. Examination of scalp-wide EEG power indicated larger alpha-range (7–13 Hz) power for the eyes closed versus eyes open conditions over posterior scalp regions. The scalp foci of these differences were near scalp sites PO7 and PO8. Mean alpha-range power at site PO7 was 37.24 dB (*SD* = 4.18), 95% *CI* [35.34, 39.14] for the eyes-closed condition versus 34.12 dB (*SD* = 3.33), 95% *CI* [32.61, 35.64] for the eyes-open condition. Mean alpha-range power at site PO8 was 37.12 dB (*SD* = 3.94), 95% *CI* [35.32, 38.91] for the eyes-closed condition versus 34.07 dB (*SD* = 3.55), 95% *CI* [32.46, 35.69] for the eyes-open condition. Statistical testing confirmed these descriptive observations (PO7: *t*(20) = 10.07, *p* < 0.001; PO8: *t*(20) = 9.35, *p* < 0.001).

#### 3.1.2. Observed EEG Signal Regularity

The run test for randomness applied to the unfiltered EEG data yielded mean *RTR* statistic values of 0.995 (*SD* = 0.023), 95% *CI* [0.005, 1.000] for the eyes-open condition and 1.000 (*SD* = 0.001), 95% *CI* [0.999, 1.000] for the eyes-closed condition. This result indicates that the scalp-level EEG signals were relatively regular in nature prior to data filtering. After filtering the EEG signals, the mean *RTR* statistic values were 1 with zero variability (*SD* = 0) for both the eyes-open and eyes-closed conditions. This indicates that additional (near-perfect) regularity was imposed onto the unfiltered EEG signals via the smoothing operation of the band-pass filter.

#### 3.1.3. Observed EEG Functional Integration and Topological Complexity

The summary statistics for the observed resting state EEG integration, *I*(*X*), and interaction complexity, *C_I_*(*X*), are given in the left columns Table 1. Nonparametric statistical tests showed that, on average, *I*(*X*) was larger for eyes-closed versus eyes-open conditions, *t*(20) = 6.84, *p <* 0.001, and *C_I_*(*X*) was smaller for eyes-closed versus eyes-open conditions, *t*(20) = −3.94, *p <* 0.001. Additionally, average *I*(*X*) and *C_I_*(*X*) were negatively correlated with each other, *R* = −0.58, *p* < 0.008.

The summary statistics for the surrogate *I*(*X*) and *C_I_*(*X*) values are given in the right columns of Table 1. All means and 95% *CIs* for observed integration and complexity lay outside the surrogate 95% two-tailed confidence intervals for both resting-state conditions. Thus, it is unlikely that the observed resting state integration and complexity are attributable to spurious volume-conducted interactions. Moreover, the surrogate *I*(*X*) values were smaller and the surrogate *C_I_*(*X*) values were larger than the observed values. This pattern can be explained in terms of a shift of the surrogate data towards lower structural order relative to the observed resting state EEG due to the randomization process utilized during the construction of the surrogate data.

#### 3.1.4. Observed EEG Brain Network Parameters

**Figure 7:**
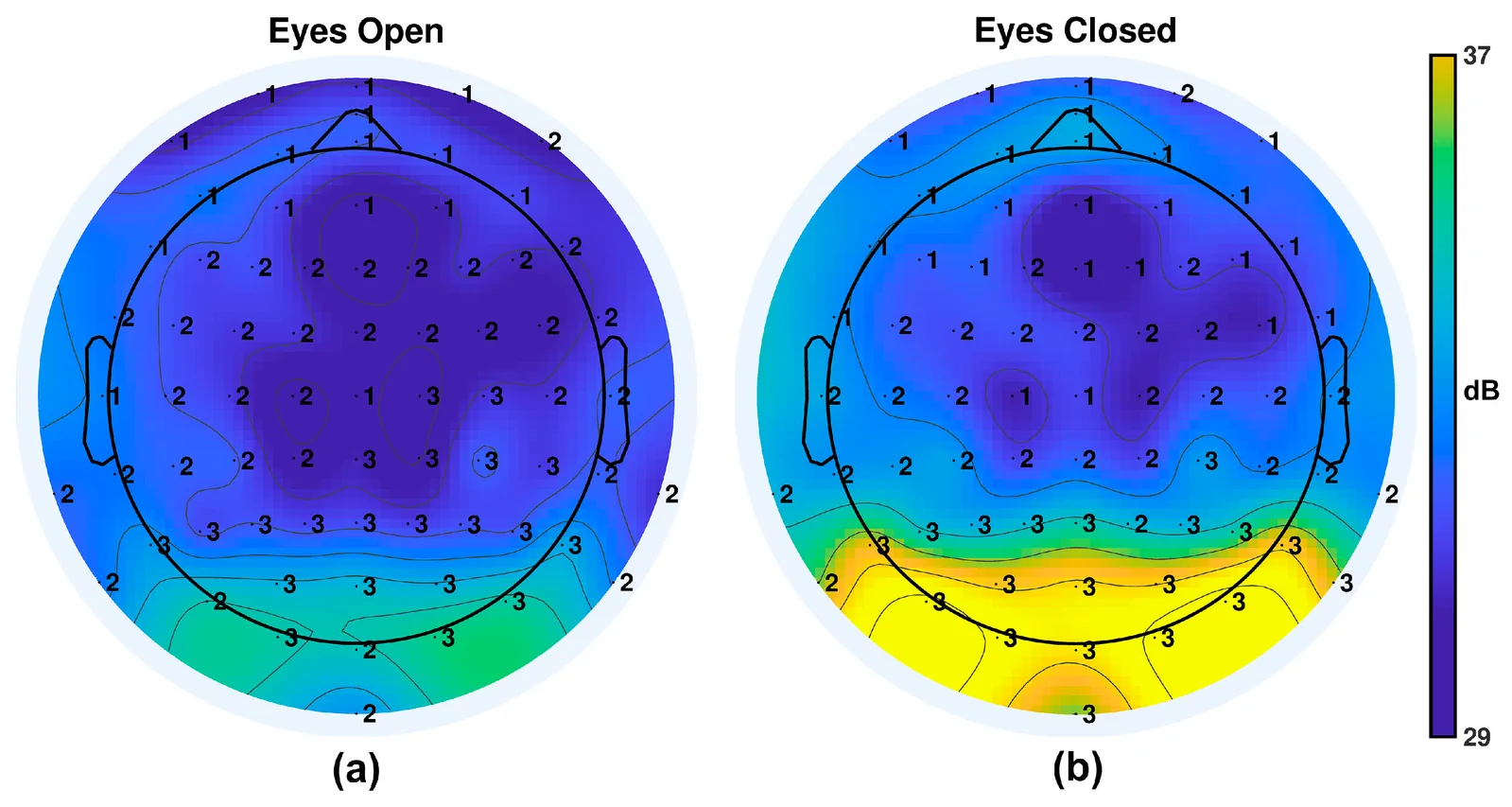
Modular partitioning of the alpha-range scalp EEG signals for the (**a**) eyes-open condition and (**b**) eyes-closed condition. Each number indicates the representative module label (e.g., Module 1, Module 2, etc.) for each electrode site. These values were determined by taking the cross-trial mode of module label numbers returned by the community detection algorithm at each electrode site for each participant and condition. The mode of these individual values across participants were then taken as the representative module label for each electrode site and condition. Head map colors display the scalp topography of the average alpha-range *PSD* for each condition. Note that the pattern of estimated modularity roughly follows the variation of *PSD* across the scalp.

Figure 7 shows the modular partitioning of the alpha-range scalp EEG signals for the eyes-closed and eyes-open conditions against the backdrop of alpha-range EEG power scalp distribution. This figure suggests that the community detection algorithm used here (see Section 2.8.2: Detection and Analysis of Brain Network Modular Structure, above) identified approximately three to four modules for each resting state condition, but the modules for the eyes-closed condition were larger and less variable in size and shape than those for the eyes-open condition.

The summary statistics for the observed resting-state EEG network parameters (Table 2, top rows) largely confirmed this pattern, as did the nonparametric inferential statistics comparing the summary EEG network parameter values between the two resting-state conditions. The number of modules *N_M_* was smaller for eyes-closed versus eyes-open conditions on average, *t*(20) = −3.22, *p* < 0.007, whereas the size of the modules *M_Size_* was greater for eyes-closed versus eyes-open conditions, *t*(20) = 3.30, *p* < 0.007. Mean modularity *Q* was larger for eyes-closed versus eyes-open conditions, *t*(20) = 3.46, *p* < 0.005, indicating a stronger community structure for the eyes-closed condition. Mean participation coefficient *PC* and mean participation index *PI* were smaller for eyes-closed than eyes-open conditions, *PC*: *t*(20) = −3.59, *p* < 0.004; *PI*: *t*(20) = −4.92, *p* < 0.001, indicating that, on average, scalp nodes were distributed across fewer modules for eyes-closed versus eyes-open conditions. Moreover, the estimated resolution parameter *γ* was smaller for eyes-closed versus eyes-open conditions. This difference, though small, was statistically significant, *t*(20) = −2.94, *p* < 0.008, with the direction of this difference consistent with the observation of fewer modules for eyesclosed versus eyes-open conditions as detected by the iterative community detection algorithm used here.

Finally, the summary statistics for the surrogate network parameter values (Table 2, lower rows) indicate that it is unlikely that the observed pattern of network parameter values are attributable to spurious volume-conducted interactions. All means and 95% *CIs* for observed network parameters lay outside the surrogate 95% two-tailed confidence intervals for both resting-state conditions.

#### 3.1.5. Relationships Between Observed EEG Network Parameters and Integration/Complexity

To examine the relationships between the observed EEG network parameters and integration and complexity, each participant’s mean values for the eyes-open condition was subtracted from the corresponding values for the eyes-closed condition to create difference scores for each measure: Δ*I*(*X*), Δ*C_I_*(*X*), Δ*Q*, Δ*PC*, Δ*PI*, Δ*N_M_*, Δ*M_Size_*, and Δ*γ*. Then, across-participant correlations were computed between Δ*I*(*X*) and Δ*C_I_*(*X*) versus each network measure difference (Table 3).

Statistically significant positive correlations were observed between Δ*I*(*X*) and Δ*Q* and Δ*M_Size_*, whereas significant negative correlations were observed between Δ*I*(*X*) and Δ*PC*, Δ*PI*, Δ*N_M_*, and Δ*γ*. These results indicate that as EEG integration increased across the eyes-open to eyes-closed conditions, the alpha-range EEG signals organized themselves into fewer, larger, and less variable (in shape and location) modules.

Statistically significant positive correlations were observed between Δ*C_I_*(*X*) and Δ*PC*, Δ*PI*, Δ*N_M_*, and Δ*γ*, whereas significant negative correlations were observed between Δ*C_I_*(*X*) and Δ*Q* and Δ*M_Size_*. These results indicate that as EEG complexity decreased across the eyes-open to eyes-closed conditions, the alpha-range scalp EEG also organized into fewer, larger, and less variable (in shape and location) modules.

### 3.2. Dipole Simulation of Resting State EEG

#### 3.2.1. Regularity of Dipole Simulations

**Figure 8:**
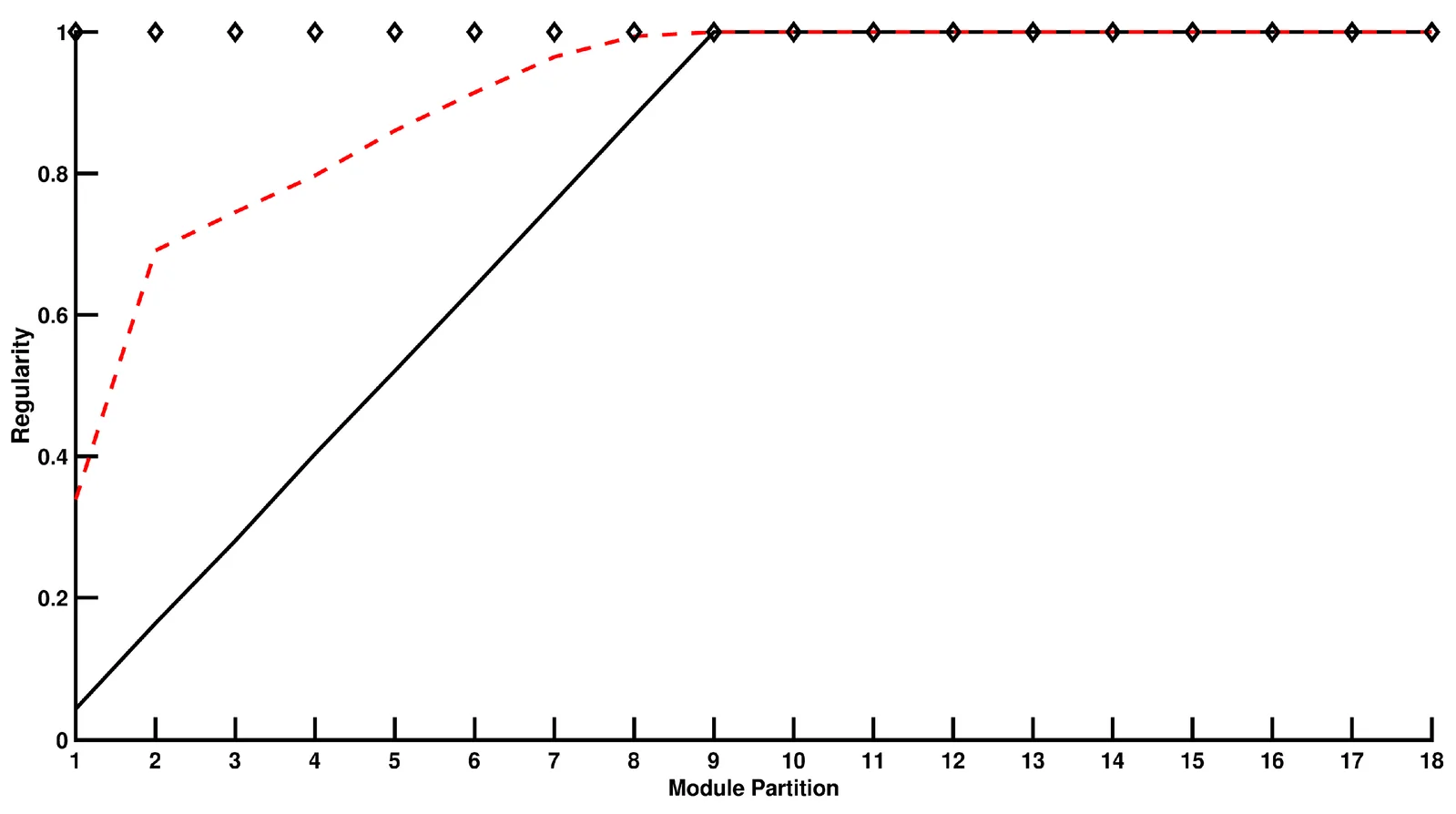
Mean dipole simulation *RTR* statistics for dipole source signals (solid black line), unfiltered scalp EEG (dashed red line), and filtered scalp EEG signals (open diamonds). Data points reflect averages across sources/electrodes and 432 simulated trials. The standard errors of the RTR statistics are too small to be displayed but ranged from 0 to 0.001 for the dipole source signals and 0 to 0.003 for the scalp EEG signals. Standard errors were 0 for the filtered scalp EEG signals, indicating near-perfect regularity.

Figure 8 shows the mean *RTR* statistic values of the run test for randomness applied to the simulated dipole source signals and the unfiltered and filtered scalp-level EEG signals for each module partition. Mean *RTR* statistic values were initially low for Module Partition 1 of both the source-level and unfiltered scalp-level signals but then gradually increased among the remaining Dipole Model Manipulation Phase 1 module partitions until reaching a maximum value of 1 at Module Partition 9, which remained constant for Module Partitions 10–18. These results indicate that the dipole sources and unfiltered scalp EEG signals became more regular as they transitioned from spatiotemporally independent aperiodic signals to temporally dependent yet (relatively) spatially independent periodic signals across the Phase 1 simulations. These signals remained regular/periodic for the Dipole Model Manipulation Phase 2 module partitions. In contrast, the mean *RTR* statistic values for the filtered scalp EEG signals had a constant value of one across all eighteen module partitions (near-perfect regularity). This indicates that additional regularity was imposed onto the unfiltered scalp EEG signals via the smoothing operation of the band-pass filter, similar to that observed for the filtered empirical scalp EEG data.

#### 3.2.2. Simulated EEG Functional Integration and Topological Complexity

**Figure 9:**
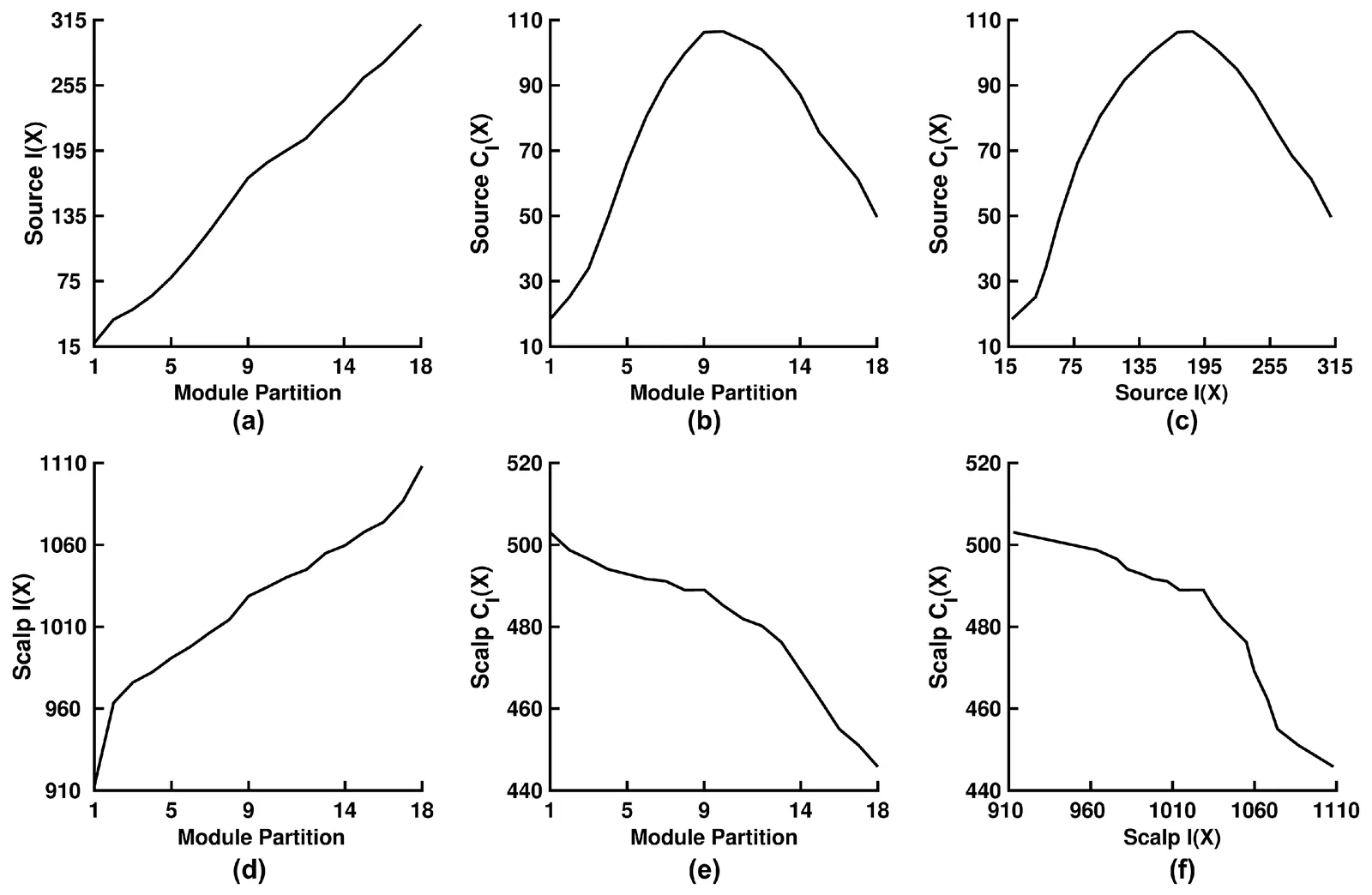
Dipole simulation integration and complexity. (**a**) Integration *I*(*X*) as a function of Module Partition (Dipole Model Manipulation Phase 1: Partitions 1–9; Phase 2: Partitions 10–18) for simulated occipital dipole sources only; (**b**) Interaction complexity *C_I_*(*X*) as a function of Phase 1 and 2 Module Partitions for simulated occipital dipole sources; (**c**) Interaction complexity *C_I_*(*X*) as a function of integration *I*(*X*) for simulated occipital dipole sources; (**d**) Integration *I*(*X*) as a function of Phase 1 and 2 Module Partitions for simulated whole-scalp EEG resulting from volume-conducted activity of all dipole sources (occipital and background dipoles); (**e**) Interaction complexity *C_I_*(*X*) as a function of Phase 1 and 2 Module Partitions for simulated scalp EEG; (**f**) Interaction complexity *C_I_*(*X*) as a function of integration *I*(*X*) for simulated scalp EEG. All values are in units of bits. Data points reflect averages across 432 simulated trials; the standard deviations of simulated *I*(*X*) and *C_I_*(*X*) are too small to be displayed but ranged from 0.04 to 0.70 bits for the source-level simulations and 0.21 to 1.55 bits for the scalp-level simulations.

Figure 9 shows the integration *I*(*X*) and interaction complexity *C_I_*(*X*) for the dipole simulations as a function of module partition. Figure 9, panels a–c show the results for the occipital dipole moment sources only (not including the background sources). Panels a and b of Figure 9 show average source-level *I*(*X*) and *C_I_*(*X*) as a function of module partition. As expected, *I*(*X*) increased across module partitions during the initial phase of the dipole simulations (Dipole Model Manipulation Phase 1: Module Partitions 1–9) in which the dipole sources initially changed from spatiotemporally independent aperiodic signals to temporally dependent yet (relatively) spatially independent periodic signals. I(X) continued to increase further across the second phase of the dipole simulations (Phase 2: Module Partitions 10–18) as the periodic signals changed from temporally dependent and (relatively) spatially independent to periodic signals with near-perfect spatiotemporal dependency. Interaction complexity *C_I_*(*X*) first increased across the Phase 1 module partitions, reaching a maximum at Partition 9 (periodic, temporally dependent, spatially independent sources) before decreasing across the Dipole Model Manipulation Phase 2 Module Partitions as the periodic dipole sources became more spatiotemporally dependent. Figure 9c shows average *C_I_*(*X*) as a function of average *I*(*X*) for the simulated dipole sources. *C_I_*(*X*) was initially low when *I*(*X*) was low, gradually increasing to a maximum when *I*(*X*) was moderately valued (corresponding to Module Partition 9 when all the dipoles were periodic, temporally dependent, and relatively spatially independent), before decreasing again to a minimum as *I*(*X*) increased further to its maximum values.

This relationship was confirmed via two correlation analyses, one quantifying the Phase 1 relationship, *R* = 0.98, *p <* 0.001, and the second quantifying the Phase 2 relationship, *R* = −0.99, *p <* 0.001. This inverted-U relationship between the simulated source-level complexity and integration agrees with theoretical predictions (Figure 1).

Figure 9, panels d–f show the results for the simulated scalp-level EEG (which now also includes a contribution from the background dipole sources). Panels d and e of Figure 9 show average scalp-level EEG *I*(*X*) and *C_I_*(*X*) as a function of module partition. *I*(*X*) in-creased across module partitions as the dipole sources initially changed from aperiodic to periodic (Dipole Model Manipulation Phase 1: Module Partitions 1–9) and as the dipole sources changed from temporally dependent and (relatively) spatially independent periodic signals to periodic signals with near-perfect spatiotemporal dependency (Dipole Model Manipulation Phase 2: Module Partitions 10–18). *C_I_*(*X*) decreased from a global maximum to a local minimum across the Phase 1 and Phase 2 module partitions. Figure 9f shows average *C_I_*(*X*) as a function of average *I*(*X*) for the simulated scalp level signals where, save for an initial increase in *C_I_*(*X*) over the initial low values of *I*(*X*), overall *C_I_*(*X*) decreased to a minimum as *I*(*X*) increased to a maximum. This general decrease in scalp-level *C_I_*(*X*) with increasing *I*(*X*) was confirmed via correlation analysis, R = −0.92, *p* < 0.001, a result in agreement with the observed *I*(*X*) and *C_I_*(*X*) relationship.

It should be noted that the scalp-level EEG functional integration and topological complexity findings shown in Figure 9 were obtained after filtering the simulated signals in the alpha range (see Section 2.4: Dipole Modeling and Simulation of Resting State EEG Data, above). However, it was shown in Section 3.2.1: Regularity of Dipole Simulations, above, that such filtering is likely to have imposed additional regularity on the scalp EEG signals that could have affected the estimates of *I*(*X*) and *C_I_*(*X*). As a check on this possibility, EEG functional integration and complexity were computed for unfiltered simulated scalp EEG signals; see Figure S1 of the Supplementary Materials. The results for scalp-level *I*(*X*) were largely unchanged from the analysis conducted on the filtered simulated scalp signals (see Figure S1a), but there was a small alteration in the pattern of scalp-level *C_I_*(*X*) across module partitions of the dipole simulations (see Figure S1b). In this case, *C_I_*(*X*) initially increased across the first three Phase 1 module partitions, peaking at Module Partition 3, before decreasing for the remaining Phase 1 partitions and the Phase 2 partitions as well. This means that *C_I_*(*X*) increased to a maximum over the initial low values of *I*(*X*), before decreasing to a minimum as *I*(*X*) increased to a maximum over low, medium, and high levels of *I*(*X*); see Figure S1c. This general decrease in scalp-level *C_I_*(*X*) with increasing *I*(*X*) was confirmed via correlation analysis, *R* = −0.96, *p* < 0.001, a result in agreement with the observed *I*(*X*) and *C_I_*(*X*) relationship.

#### 3.2.3. Simulated EEG Network Parameters

Table 4 summarizes the general observations of the EEG network parameters for the source-level and scalp-level simulations. Detailed explications of these observations are given in the sections Source-Level Simulations and Scalp-Level Simulations, below.

##### Source-Level Simulations

As an initial qualitative assessment, the true modular partitioning of the simulated alpha-range occipital dipole moment sources was visually compared with the estimated module partitioning obtained from the community detection algorithm used here (see Section 2.8.2: Detection and Analysis of Brain Network Modular Structure, above). This assessment is shown in Figure S2 of the Supplementary Materials. It is clear from the figure that the community detection algorithm accurately estimated the module partitions only when they consisted of four modules or fewer (Module Partitions 15–18), whereas the algorithm underestimated the size and number of modules for the remaining partitions (see Section 4.6: Study Limitations, below, for discussion about the limits of the present modularity estimation procedure). Nevertheless, the algorithm correctly estimated the direction of change in size and number of the modules for each partition across the two dipole manipulation phases of the simulations. Across Dipole Model Manipulation Phase 1, where the dipole sources initially changed from spatiotemporally independent aperiodic signals to periodic signals that were temporally dependent and (relatively) spatially independent, the number and size of the estimated modules remained relatively constant. However, during Dipole Model Manipulation Phase 2, in which the periodic signals changed from temporally dependent and (relatively) spatially independent to periodic signals with near-perfect spatiotemporal dependency, the estimated modules grew larger in size and their number decreased.

**Figure 10:**
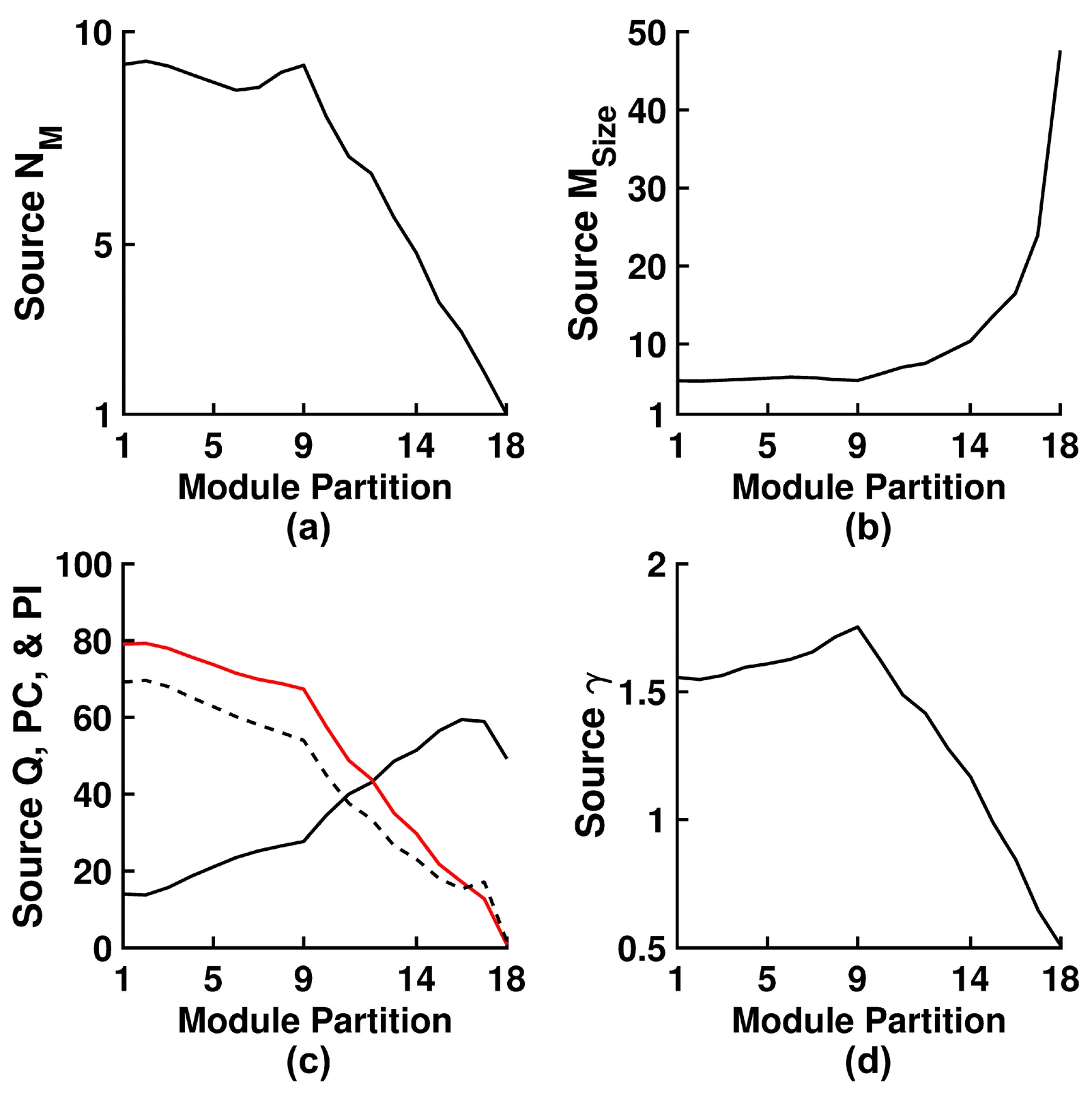
Source-level dipole simulation network parameters as a function of module partition. (**a**) Total number of modules per partition *N_M_* as a function of Phase 1 and 2 module partitions (Dipole Model Manipulation Phase 1: Partitions 1–9; Phase 2: Partitions 10–18); (**b**) Module size *M_Size_* as a function of Phase 1 and 2 module partitions; (**c**) Modularity *Q* (solid black/dark line), participation coefficient *PC* (red/light line), and participation index *PI* (dashed line), as a function of Phase 1 and Phase 2 module partition; (**d**) Source-level *γ* parameter as a function of Phase 1 and 2 module partitions. *Q*, *PC*, and *PI* values are expressed as percentages of the original 0 to 1 range of these measures to more easily report small differences across conditions; the remaining network parameters are dimensionless. Data points reflect averages across 432 simulated trials; the standard errors of simulated measures are too small to be displayed but ranged from 0.09 to 2.47% for source-level *Q*, *PC*, and *PI*; 0.01 to 0.18 for source-level *N_M_*; 0.06 to 0.28 for source-level *M_Size_*; 0.004 to 0.024 for source-level *γ* parameter.

This qualitative assessment was confirmed quantitatively via the estimated network parameters for the simulated EEG dipole sources (Figure 10). As shown in the figure, the total number of modules per partition *N_M_* for the dipole sources (Figure 10a) and the mean size of the modules *M_Size_* (Figure 10b) both fluctuated near a constant value across Dipole Manipulation Phase 1 module partitions before steadily decreasing across the Dipole Manipulation Phase 2 module partitions. The increase for *M_Size_* accelerated rapidly beyond Module Partition 14.

Moreover, the participation coefficient *PC* and participation index *PI* for the dipole sources decreased from maximum to minimum levels across both dipole manipulation phases (Figure 10c). This indicates that as the EEG sources transitioned from spatiotemporally independent aperiodic signals (Module Partition 1) to independent periodic signals (Module Partition 9) to periodic signals with near-perfect spatiotemporal dependency (Module Partition 18), each source node became less connected to nodes in other modules and became more connected to nodes within a single module.

Furthermore, the modularity statistic *Q*, which quantifies the degree to which a network can be partitioned into non-overlapping groups of nodes, increased across both dipole manipulation phases (Figure 10c), reaching a peak at Module Partition 16 before decreasing slightly over Module Partitions 17 and 18. This indicates the module partitions became “stronger” with more clearly delineated source node communities across Dipole Manipulation Phases 1 and 2.

Finally, the resolution parameter *γ* (Figure 10d) initially increased from a minimum across the Phase 1 module partitions to reach a maximum at Module Partition 9, before decreasing to a second minimum across the Phase 2 module partitions. This indicates a changing bias in EEG dipole source node community detection from smaller communities to larger communities, consistent with the change in module size across dipole manipulation phases.

##### Scalp-Level Simulations

As an initial qualitative assessment, the estimated EEG PSD and modular partitioning of the simulated scalp-level alpha-range EEG signals was visualized to compare with the modular partitioning of empirical EEG data. This visualization is shown in Supplementary Materials Figure S3. This figure shows that EEG PSD increased in intensity across Phase 1 to Phase 2 module partitions, with the scalp-wide distribution of PSD exhibiting a similar pattern to that observed for the observed EEG PSD. However, the simulated scalp EEG modularity patterns did not exactly match those of the observed data. None of the estimated module partitions from the simulations exactly matched the estimated pattern of modular partitioning of the empirical scalp-level data, which appeared to be somewhere in between that of Module Partitions 17 and 18 of the simulated scalp-level data (a conclusion consistent with the EEG network parameter results reported below); see Figure S3. This difference may have arisen from modularity maximization errors or imperfections of the present dipole source moment model, an issue discussed further in Section 4.6: Study Limitations, below. Nevertheless, the pattern of changes in scalp-level modular partitioning was like that of the empirical scalp EEG signals in that that the modules grew larger, became fewer in number, and became less variable in shape and location across the Phase 1 to Phase 2 module partitions. 

**Figure 11:**
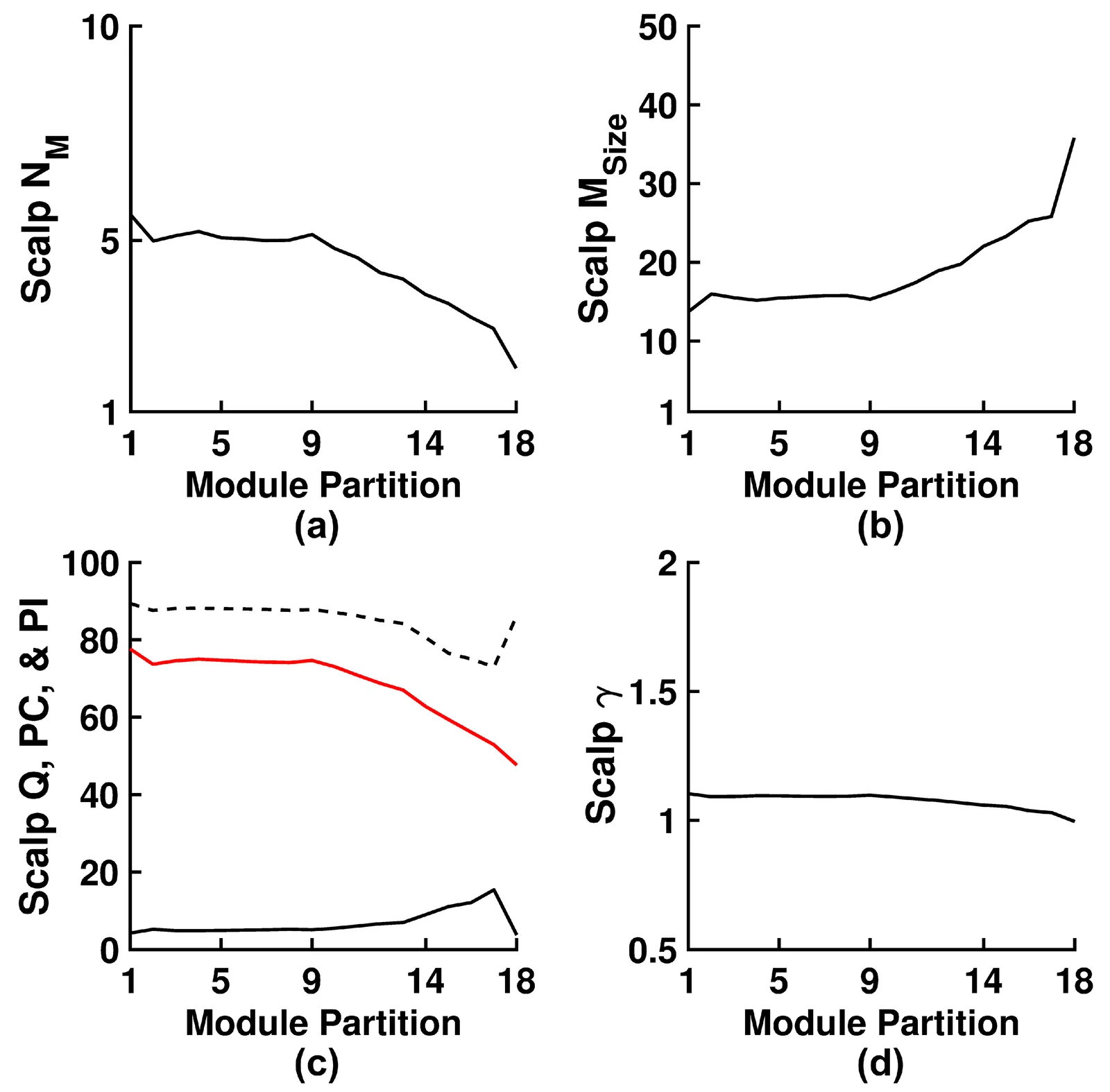
Scalp-level dipole simulation network parameters as a function of module partition. (**a**) Total number of modules per partition *N_M_* as a function of Phase 1 and 2 module partitions (Dipole Model Manipulation Phase 1: Partitions 1–9; Phase 2: Partitions 10–18); (**b**) Module size *M_Size_* as a function of Phase 1 and 2 module partitions; (**c**) Modularity *Q* (solid black/dark line), participation coefficient *PC* (red/light line), and participation index *PI* (dashed line), as a function of module partition; (**d**) Scalp-level *γ* parameter as a function of Phase 1 and 2 module partitions. *Q*, *PC*, and *PI* values are expressed as percentages of the original 0 to 1 range of these measures to more easily report small differences across conditions; the remaining network parameters are dimensionless. Data points reflect averages across 432 simulated trials; the standard errors of simulated measures are too small to be displayed but ranged from 0.07 to 1.31% for scalp-level *Q*, *PC*, and *PI*; 0.005 to 0.189 for scalp-level *N_M_*; 0.05 to 0.86 for scalp-level *M_Size_*; and 0.0003 to 0.0066 for scalp-level *γ* parameter.

The qualitative assessment of the scalp-level modular partitioning was consistent with the quantitative assessment obtained via the estimated network parameters for the simulated scalp EEG signals (Figure 11). As shown in the figure, the total number of modules per partition *N_M_* for the scalp EEG (Figure 11a) fluctuated near a constant value across Dipole Manipulation Phase 1 module partitions before steadily decreasing across the Dipole Manipulation Phase 2 module partitions. The mean size of the scalp modules *M_Size_* (Figure 11b) also fluctuated near a constant value across Phase 1 module partitions before steadily increasing across Phase 2 module partitions. Moreover, the participation coefficient *PC* for the simulated scalp EEG decreased from maximum to minimum levels across both dipole manipulation phases (Figure 11c). The participation index *PI* (Figure 11c) also decreased across Phase 1 and most of Phase 2 but increased slightly from Module Partition 17 to Module Partition 18. These overall changes in *PC* and *PI* were like those observed for the EEG dipole sources and indicate that each simulated scalp EEG signal node became less connected to nodes in other scalp-level modules and became more connected to nodes within a single module.

The scalp-level modularity statistic *Q* increased across both dipole manipulation phases (Figure 11c), reaching a peak at Module Partition 17 before decreasing slightly to Module Partition 18. These changes indicate that the scalp-level module partitions became “stronger” with more clearly delineated node communities across Dipole Manipulation Phase 1 and 2, again similar to the change in Q observed for the EEG dipole sources.

The resolution parameter *γ* (Figure 11d) stayed at a constant value (slightly larger than *γ* = 1) across both phases of the dipole simulations before slightly decreasing across Module Partitions 17 and 18. This indicates that the community detection algorithm became tuned to detect intermediate-size scalp node communities.

#### 3.2.4. Relationships Between Simulated EEG Network Parameters and Integration/Complexity

To examine the relationships between the EEG network parameters and the integration and complexity of the simulated dipole moments, across-partition linear correlations were computed between each network measure and *I*(*X*) and *C_I_*(*X*) (Table 5). Due to the non-monotonic inverted-U shape of the source-level *C_I_*(*X*), correlations involving this measure were performed separately for the Phase 1 and Phase 2 module partitions. Otherwise, the correlations were performed across the entire range of module partitions (Module Partitions 1–18) for the remaining dipole simulation measures (source- and scalp-level *I*(*X*), scalp-level *C_I_*(*X*)).

For both the source- and scalp-level simulations, statistically significant positive correlations were observed between *I*(*X*) and *Q* and *M_Size_*, whereas significant negative correlations were observed between *I*(*X*) and *PC*, *PI*, *N_M_*, and *γ*. These results indicate that as EEG integration increased across module partitions, the simulated source-level and scalp-level EEG signals organized themselves into fewer, larger, and less variable (in shape and location) modules.

For the Phase 1 source-level simulations, statistically significant positive correlations were observed between *C_I_*(*X*) and *Q* and *γ*, whereas significant negative correlations were observed between *C_I_*(*X*) and *PC* and *PI*. An opposite pattern resulted in the Phase 2 source-level simulations, with significant positive correlations observed for *C_I_*(*X*) and *PC*, *PI*, *N_M_*, and *γ*, whereas significant negative correlations were observed between *C_I_*(*X*) and *Q* and *M_Size_*. These results indicate that as source-level EEG complexity first increased across the Phase 1 module partitions and then decreased across the Phase 2 module partitions, the simulated source-level EEG signals organized into fewer, larger, and less variable (in size and shape) modules.

Finally, across both phases of the scalp-level simulations, significant positive correlations were observed between *C_I_*(*X*) and *PC*, *PI*, *N_M_*, and *γ*, whereas significant negative correlations were observed between *C_I_*(*X*) and *Q* and *M_Size_*. In general, these results indicate that as simulated EEG complexity first increased across the Phase 1 module partitions and then decreased across the Phase 2 module partitions, the simulated EEG scalp signals organized into fewer, larger, and less variable (in size and shape) modules. This was particularly the case across the Phase 2 module partitions.

## 4. Discussion

### 4.1. Source- vs. Scalp-Level Resting State EEG Functional Integration and Topological Complexity

The goal of the present study was to understand the previously observed deviation of the functional integration–complexity relationship for scalp-level EEG signals from the theoretically-predicted curvilinear inverted-U relationship observed (using simulations) at the level of cortical dipole EEG sources [23,24]. This discrepancy between the source- and scalp-level relationships was replicated in the present study within simulated and empirically measured data. EEG dipole source simulations showed that EEG functional integration and topological complexity followed an inverted-U relationship at the level of the cortex; as *I*(*X*) increased among the dipole sources, *C_I_*(*X*) initially increased from a local minimum to a global maximum during Dipole Model Manipulation Phase 1 of the simulations when the dipole sources initially changed from spatiotemporally independent aperiodic signals to temporally dependent, yet (relatively) spatially independent, periodic signals (see Section 2.4: Dipole Modeling and Simulation of Resting State EEG Data, above). Then as *I*(*X*) for the dipole sources increased further, *C_I_*(*X*) decreased to a second local minimum during Dipole Model Manipulation Phase 2 of the dipole simulations when the periodic signals changed from temporally dependent and relatively spatially independent to periodic signals with near-perfect spatiotemporal dependency (see Section 2.4: Dipole Modeling and Simulation of Resting State EEG Data, above).

However, when the source EEG signals were projected to the scalp, EEG functional integration and topological complexity followed a monotonic inverse relationship, with *C_I_*(*X*) decreasing from a global maximum to a local minimum as *I*(*X*) increased among the scalp EEG signals (and the cortical dipole sources). The empirical scalp EEG signals displayed a similar monotonically decreasing relationship between *I*(*X*) and *C_I_*(*X*) in the transition from the eyes-open resting state (medium integration, high complexity) to the eyes-closed resting state (high integration, low complexity). This observation is consistent with the placement of the empirical EEG data in the mid- to high-integration regime (Dipole Model Manipulation Phase 2 of the dipole simulations; see Section 2.4: Dipole Modeling and Simulation of Resting State EEG Data, above) of the functional integration–complexity relationship, as initially proposed by [24]. These empirical observations were accompanied by larger EEG power for the eyes closed than eyes open resting state conditions, a pattern observed by multiple previous studies [23,24,29,78,79]. However, these empirical integration and topological complexity findings should be unaffected by this EEG power difference because the information theoretic *I*(*X*) and *C_I_*(*X*) measures used here involve entropy differences and thus are scale-independent [20,24,80].

This study explored three different factors—volume conduction, EEG signal regularity, and brain modularity—that could affect the measurement and quantification of EEG functional integration and complexity, and thus potentially account for the discrepancy between the source- and scalp-level EEG functional integration–complexity relationships. All three factors were explicitly manipulated in the simulations (see Section 2.4: Dipole Modeling and Simulation of Resting State EEG Data, above) and formally assessed for both the simulated and empirical EEG data (see Methods Section 3.1: Observed EEG Data and Section 3.2: Dipole Simulation of Resting State EEG, above). The findings for each factor are discussed in more detail below.

### 4.2. Effect of Volume Conduction

The spatial mixing effects of volume conduction were explicitly manipulated in the dipole simulations (see Section 2.4: Dipole Modeling and Simulation of Resting State EEG Data, above) by projecting the EEG dipole source signals through the head from the model cortex to the model scalp. This resulted in the discrepancy between the source- and scalp-level EEG functional integration–complexity relationships observed in previous studies; whereas source-level EEG functional integration and topological complexity followed a curvilinear “inverted-U” relationship, scalp-level EEG functional topological complexity followed an inverse relationship to functional integration. It should be noted that this spatial mixing of cortical EEG signal occurred even though the scalp-level EEG signals were transformed into Laplacian-based current source densities to reduce the effects of volume conduction on the integration, complexity, and network measures (see Section 2.3: EEG Recording and Preprocessing, above). Laplacian-transformed EEG signals are less prone to across-electrode contamination of activity from a single physical scalp location and thus provide a clearer spatial representation of the cortical source distributions underlying scalp EEG topographies (at least for superficial EEG sources) [33,36,37,81,82]. Indeed, these beneficial effects of the Laplacian transformation were demonstrated by [24], who found that resting-state scalp EEG functional topological complexity was enhanced, and functional integration was reduced, when using the Laplacian transformation versus other EEG reference schemes. Nevertheless, like in the present study, the scalp-level distortion of the EEG functional integration–complexity relationship was observed for all EEG reference schemes [24]. This suggests that measures to sharpen the spatial distribution of scalp EEG signals, though recommended, cannot completely remove the distortive effects of volume conduction on EEG functional integration and topological complexity. However, this distortion may be potentially eliminated if integration and complexity analysis were to be performed directly on estimated EEG cortical sources obtained through use of various EEG source estimation techniques in combination with machine learning, as recently suggest by [34]. This is a topic for future research.

### 4.3. Effect of Signal Regularity

EEG signal regularity was also explicitly manipulated in the simulations (see Section 2.4: Dipole Modeling and Simulation of Resting State EEG Data, above) and statistically assessed for both the simulated and empirical EEG data (see Section 2.6: Computation of EEG Signal Regularity, above). EEG regularity increased during Phase 1 of the simulations (parameterized as an increasing proportion of periodic dipole sources across module partitions) and then stayed constant for Dipole Model Manipulation Phase 2 of the simulations (where all sources were periodic but became more synchronized across module partitions). These regularity patterns, in conjunction with the inverted-U integration–complexity relationship for the dipole sources, indicate that the activity of the brain (or of any other system with elements that can engage in periodic or aperiodic behavior) is most complex when the time courses of its neural elements are periodic but otherwise spatially independent of each other. It is unclear whether this conclusion should generalize to other kinds of topologies (e.g., from physical space of electrodes to general state space). This is a topic for future research.

However, the distorted scalp-level functional integration–complexity relationship suggests that the regularity of the EEG signals at the scalp is different from that of the source signals. One likely reason is the filtering of the scalp-level EEG signals in the alpha-band (7–13 Hz) range. The present analysis focused on this frequency range following previous studies of EEG functional integration and topological complexity [23,24,26,28], because of its predominance during wakeful resting states [29] and evidence that information–theoretic computations may be more accurate when performed on data with a narrower frequency range [28]. This filtering resulted in constant-regularity scalp EEG signals across Dipole Model Manipulation Phases 1 and 2 of the simulations, whereas the regularity of unfiltered scalp EEG signals increased in a similar manner to the dipole sources (see Section 3.2.1: Regularity of Dipole Simulations, above). Nevertheless, it is unlikely that this filtering was solely responsible for the distortion of the scalp-level EEG functional integration–complexity relationship, because this relationship for the unfiltered scalp EEG signals displayed a similar distortion as the filtered signals (see Results Section 3.2.2: Simulated EEG Functional Integration and Topological Complexity, above, and Figure S1 in the Supplementary Materials). This suggests that spatial mixing due to volume conduction was the main reason for the distortions of the scalp-level EEG functional integration–complexity relationship investigated here, but that such mixing also affected the regularity of the scalp-level signals. This conclusion is also supported by the observation that the regularity of the unfiltered EEG scalp signals was greater than the regularity of the EEG sources, a result that could occur in the present simulations only due to the mixing effect of volume conduction.

Another observation of note is that the alpha-band filtered and unfiltered empirical scalp EEG signals were both highly periodic (see Section 3.1.2: Observed EEG Signal Regularity, above). Given the present simulation results, this indicates that the resting-state empirical EEG signals inhabited the high integration regime approximately captured by the Dipole Model Manipulation Phase 2 module partitions of the dipole simulations, confirming the speculation of [24]. Thus, the present findings illustrate the utility of regularity assessment of unfiltered scalp EEG signals when interpreting the scalp-level EEG complexity. If scalp EEG signals reflect predominantly aperiodic sources in the lower integration regime (to the left of the complexity peak depicted in Figure 1), they should demonstrate statistically significant departures from regularity, whereas if the signals reflect periodic sources in the higher integration regime (to the right of the complexity peak depicted in Figure 1), they should not.

### 4.4. Effect of Modularity

EEG dipole source modular organization was manipulated in the simulations (see Section 2.4: Dipole Modeling and Simulation of Resting State EEG Data, above) and statistically assessed for both the simulated and empirical EEG data (see Section 2.8 Estimation of Brain Network Organization, above). EEG modularity was not directly manipulated during Dipole Model Manipulation Phase 1 of the simulations. Here, the regularity of an increasing proportion of dipole sources changed from aperiodic to periodic across module partitions, but there was no explicit manipulation of the dipole’s frequency and synchronization to simulate functional connectivity between sources reflective of module formation. Functional connectivity reflects statistical dependencies between nodes, rather than direct anatomical connections (i.e., structural connectivity) or directed causal relationships (i.e., effective connectivity) [27]. As such, functional connectivity is highly susceptible to random uncorrelated behavior as well as indirect effects of common inputs into two otherwise unrelated nodes. Therefore, the estimated modularity during Phase 1 of the simulations should reflect statistically spurious coincident activity between nodes. This spurious modularity was reflected in the statistical estimation of the source-level modular organization, with larger numbers of smaller detected “modules” (indicated by high *N_M_* and low *M_Size_*) that varied in shape and size (as indicated by changes in the *γ* resolution parameter, as well as the module distribution patterns of Figure 10), and with nodes that were more uniformly distributed across all “modules” rather than interaction exclusively with nodes in their own modules (indicated by high *PI* and *PC*). A small *Q* statistic indicated weak division into separate distinct network communities across module partitions. These network metrics either fluctuated around a relatively constant level or changed minimally across the module partitions of the Dipole Model Manipulation Phase 1 simulations. This contrasts with the rapid increase in topological complexity of the dipole sources during Phase 1 of the simulations, suggesting that the modularity of the sources was unrelated to their complexity, the complexity of the scalp-level EEG signals, or the discrepancy between the source- and scalp-level EEG functional integration–complexity relationships.

During Dipole Model Manipulation Phase 2 of the simulations, however, EEG modularity was directly manipulated by adjusting the synchronization and spatiotemporal dependence among the dipole sources to simulate functional connectivity and clustering. Different frequency and phase relationships corresponded to different modules; the highest degree of modular organization started at Module Partition 9 (each individual dipole source was effectively an individual module), and this then decreased across Dipole Model Manipulation Phase 2 of the simulations which involved increasing the size of the dipole source modules while simultaneously decreasing their number. These modularity manipulations were reflected in the statistical estimation of the modular organization of the dipole sources, with their modular organization reduced across the Phase 2 module partitions. Estimated modules grew larger (indicated by increases in *M_Size_*) and decreased in number (indicated by decreases in *N_M_*), were less connected with other modules and more connected within their own module (indicated by decreases in *PI* and *PC*), and were less variable in size and shape (as indicated by changes in the *γ* resolution parameter and the module distribution patterns of Figure 10; see also Figure 7 and Figure 11 for empirical and simulated scalp-level distribution patterns). A cross-partition increase in the *Q* statistic indicated an increasing strength of division into separate distinct network communities across module partitions. Interestingly, the basic pattern of modular organization manipulated for the dipole sources (Figure 10 and Supplementary Materials Figure S2) was also present at the scalp level in the dipole simulations, as indicated by similar changes in the network analysis parameters and scalp-level module distribution patterns (Figure 11 and Supplementary Materials Figure S3). However, this should not be surprising given that the *ciPLV* metric used to estimate internodal connectivity mitigated the effects of volume conduction [56]; thus, scalp-level estimates of the network parameters should follow similar patterns to estimates at the source level.

These source- and scalp-level modularity patterns, in conjunction with the inverted-U integration–complexity relationship for the dipole sources across the module partitions, suggest that the brain is the most complex when its activity is regular/periodic and highly modular (i.e., when the brain is organized into many neuronal partitions that are otherwise spatially independent of each other). Then, as its modular organization decreases, the brain’s activity becomes more integrated and less complex. This conclusion is also consistent with the estimated modular organization of the empirical scalp EEG signals. As EEG integration increased and complexity decreased across the eyes-open to eyes-closed conditions, the alpha-range EEG signals organized themselves into fewer, larger, less variable (in size and shape; see Figure 7), and less interconnected modules. These results indicate that the eyes-closed resting state is characterized by a less modular state of the brain than the eyes open condition, a conclusion consistent with previous findings that the cortex transitions from a globally connected synchronized state to a desynchronized state characterized by smaller-scale locally connected networks [83,84,85,86]. These findings emerged in the context of larger EEG power for eyes-closed than eyes-open conditions. Nevertheless, the *ciPLV* connectivity measure used to estimate modularity is from a family of connectivity metrics that are relatively insensitive to amplitude [69,70] and thus, it is unlikely that this EEG power difference contributed to these findings.

### 4.5. Dynamics of EEG Functional Integration and Topological Complexity

The present study adds to accumulated evidence that different resting EEG states reflect different modes of brain organization. Here, the eyes-closed brain state was more integrated and less topologically complex than the eyes-open state, as previously observed [23,24]. This finding is consistent with evidence that when visual information processing is disengaged with the closing of the eyes, alpha-range oscillatory pacemaker signals project from the thalamus to drive most of the visual cortex [42,78,87]. The is accompanied by the formation of large-scale modules across the visual cortex, consisting of neurons in a highly interdependent state. However, when an individual opens their eyes to engage in visual processing, the brain dynamically organizes itself into a more topologically complex state consisting of multiple distributed modules composed of oscillating neurons that interact more strongly within the module than with the other modules of the brain [4,6]. These two brain states reflect distinct points on the integration–complexity relationship curve (Figure 1), to the right of the point of optimum complexity. The latter reflects brain elements in a state of regular (periodic) activity that are otherwise independent of each other. The actual value of that complexity peak will vary based on task processing requirements or idiosyncratic factors of an individual’s brain.

These resting state findings suggest that the integration–complexity relationship in the brain changes depending on its information processing load. Similar changes in the integration–complexity relationship were previously observed in the context of a simple visual perception task [23]. In that study, visual processing load was lower at a pre-stimulus baseline (an active attentional state with no visual stimulation) relative to a post-stimulus period where visual processing was engaged. EEG topological complexity was high and integration was low during the pre-stimulus period, with an increase in integration and a decrease in complexity during visual stimulation. These changes are also consistent with the brain inhabiting the medium-to-high-integration state to the right of the peak of the integration–complexity curve, as observed for the two conditions of the resting state. Importantly, ref. [23] found that topological complexity was higher and integration was lower in both the pre- and post-stimulus conditions of the visual perception task versus eyes-open and eyes-closed conditions of a resting-state task performed by the same individuals. In the context of these previous findings, the present results suggest that if neural sources are regular (an assumed state of the brain’s normal operation), then, the brain’s integration and topological complexity will always have an inverse relationship to each other, and the brain will never exceed the maximum peak topological complexity when its sources are maximally independent of each other. From a practical perspective, what this means is that the discrepancy between the source- and scalp-level integration–complexity relationships should not pose a problem for EEG studies of the normal operation of healthy human brains, as this discrepancy occurs only for irregular EEG sources.

### 4.6. Study Limitations

One limitation of the present study is that the functional connectivity implemented among the nodes of the dipole source simulations used here was phenomenological rather than mechanistic in nature. The interactions through which interdependent relationships among neuronal generators form network modules were not directly simulated; instead, the modular brain organization resulting from such interactions was simulated (see Section 2.4: Dipole Modeling and Simulation of Resting State EEG Data, above). Moreover, the modular networks simulated here are among several kinds of networks with different functional connectivity characteristics other than pure modularity [3]. The present study focused on modular network structure because of past research showing that this is an important driver of the brain’s global network dynamics and functional integration–segregation balance [3,17]. Nevertheless, the use of other network types is unlikely to change the conclusion that the discrepancy between the source- and scalp-level EEG functional integration–complexity relationships is unrelated to the spatial organization of the dipole sources, as this discrepancy only occurred when there was spurious spatial organization of the sources due to their aperiodicity (see Section 4.4: Effect of Modularity, above). It could be the case that other network structures might increase the simulated functional topological complexity above the maximum observed here when regular/periodic brain activity is highly modular (Module Partition 9 in the present simulations). For example, one study [17] introduced a synthetic hierarchical network model combining modular organization with rich-club forming hubs that hosted more complex dynamics than other widely used benchmark networks. Such a network implemented via dipole sources is likely to display greater *C_I_*(*X*) than the simple model without the rich-club hub overlay used here, provided the regularity of the network signals is periodic (see Section 4.3: Effect of Signal Regularity, above). However, as the modularity and rich-club hub characteristics of the network are diminished, then, functional integration should increase and functional complexity should decrease; if this theoretical relationship is universal, a similar relationship should then also be observed at the scalp. Testing this prediction is a topic for future research.

A second limitation of the present study is that the cortical location, orientation, and depth of the simulated dipole sources were rough approximations to the neuronal generators typically activated during wakeful resting states. The goal here was to simply emulate general coherent parieto-occipital alpha-range cortical activation in a manner that would roughly reproduce the global patterns of resting state EEG properties observed at the scalp. One approximation of the present models is the use of the same fixed maximal amplitude for all the source and background noise dipoles (see Section 2.4.2: Simulation of Functional Modularity, above). Although there is likely a distribution of maximal amplitudes across sources in the actual cortex (and varying amplitude distributions for the cortices of different individuals), differences in dipole strengths across individuals should minimally affect the present results. This is because the information-theoretic functional integration and topological complexity measures used here are independent of scale (e.g., amplitude) [20,24,80], whereas the *ciPLV* connectivity measure used to estimate scalp EEG modularity is unaffected by amplitude [69,70]. A second approximation of the present model was the use of radial dipole sources even though the resting state activates radial and tangential dipole sources located in the cortical gyri and sulci, respectively [43]. This choice was made because scalp EEG is predominantly sensitive to radial sources and because superficial, radial sources were likely to be emphasized in the analysis of the empirical scalp EEG signals by the application of a Laplacian transformation during EEG pre-processing to reduce the effects of volume conduction [43] (see Section 2.3: EEG Recording and Preprocessing). A third approximation used in the present model is that the dipole sources in the present models were placed in a regular grid-like array 2 mm below the posterior surface of the model cortex (see Figure 3), rather than in an irregular curvilinear geometrical arrangement characteristic of actual cortical sources. More realistic configurations of dipole orientation, location, and depth would alter the basic pattern of volume-conducted correlations between scalp sensors, which in turn would alter the absolute values of mean integration and complexity and the modularity patterns detected at the scalp. Nevertheless, if the EEG source configuration stays fixed (a reasonable assumption for the resting state), then, the relative changes in these properties across conditions should follow the same general pattern observed here, especially because the *ciPLV* metric should screen out most additional volume-conducted inter-electrode correlations at the scalp that are accentuated by a realistic irregular source configuration.

Nevertheless, no effort was made to explicitly reproduce additional known neurophysiological characteristics of the resting state, such as thalamo-cortical looping [42,78], the default mode network [88,89,90], or the somatosensory-related anterior alpha-band mu rhythm [91]. The use of an imperfect dipole model may have led to some discrepancies between the modeled and empirical scalp-level data. For example, the scalp topography of the simulated EEG signals (see Figure 11) generally matched the empirical data (see Figure 6 and Figure 7), but the observed patterns of modular partitioning mismatched to a small degree, with the empirical scalp-level EEG module partition pattern (see Figure 7) placed somewhere in between the patterns observed for Module Partitions 17 and 18 of the simulated scalp-level data (see Figure 11). Otherwise, the overall patterns of integration, complexity, and statistical metrics of network community structure of the simulated data were generally consistent with those of the empirical EEG data. Furthermore, the source-level simulations presented here were unaffected by the biophysical dipole parameters, as they relied only on the temporal activity of the sources assessed prior to volume conduction and how this reflected spatial correlations among the EEG sources. Nevertheless, although future studies should utilize better models of the wakeful resting state, it is likely that the present findings will generalize to other tasks that engage ongoing EEG activity (such as mental arithmetic, reading, motor grasping, working memory, or vigilant attention tasks).

A third limitation of the present study was the focus on the EEG functional integration and topological complexity of alpha-range EEG activity. Alpha-range activity was the focus here because of its predominance during wakeful resting states [29]. One drawback of this focus was already raised for the case of assessing EEG signal regularity (see Section 4.3: Effect of Signal Regularity, above). However, another drawback is that other EEG frequency ranges, such as theta band (4–7 Hz) and beta band (14–30 Hz), also reliably correlate with resting state brain network activity [88,89,90]. Future research should examine the EEG functional integration and topological complexity of brain networks operating in these other functionally relevant frequency ranges.

A fourth limitation of the present study was the use of modularity maximization as the community detection algorithm when estimating the modular partitions of EEG-related neuronal activity (see Section 2.8.2: Detection and Analysis of Brain Network Modular Structure, above). Modularity maximization has resolution limits that arise from a bias against the detection of smaller communities, while also suffering from a degeneracy problem in which multiple possible partitions of a network yield similar maximal modularity [27]. These resolution and degeneracy concerns were addressed in the present study by using a nested three-level iterative implementation of the community detection algorithm that included a tunable resolution parameter to determine the topological scale of community detection. The tuning of this parameter was automatically optimized using a maximum likelihood-based method [66]. The degeneracy problem was further addressed by use of consensus-based clustering, which identified a representative summary of the modular structure across a set of individual partitions [27]. However, these methods of mitigating these resolution and degeneracy issues have important weaknesses. The maximum likelihood-based method used to tune the resolution parameter assumes that all module partitions have the same levels of within-module and between module connectivity. This was the case for the source-level partitions of the dipole simulations; however, it is unlikely that this requirement was met for the scalp-level module detection (simulated and empirical), due to the mixing effects of volume conduction. The consensus-based clustering method used to address the degeneracy problem can produce only a representative summary of identified individual partitions, and it is possible that a sampling algorithm could miss relevant partitions that would then bias the representative summary in an unknown way. This issue was likely to have been minimized in the present study because the community detection algorithm used here implemented many samplings across 20 different runs of the three iterative levels of the algorithm.

Nevertheless, the existence of such a partition representation bias remains a distinct possibility. Indeed, the modularity estimation procedure used in the present study did produce errors at times by underestimating the size and number of modules at the source level of analysis when the actual number of modules was greater than four (see Supplementary Materials Figure S2). Moreover, the procedure reached a resolution limit of approximately 9 to 10 modules for Module Partitions 1–9 when theoretically, the number of modules should be equal to the number of sources (*N_S_* = 48). This observation may also reflect the statistical limits of estimating functional connectivity in general, as uncorrelated signals will always show some weak spurious relationships that may drive the modularity estimation procedure (see Section 4.4: Effect of Modularity, above). However, these resolution errors were small, and their presence is unlikely to have affected the main findings and general conclusions of the present study. The use of the specific community detection methods in the present study was a simple matter of expediency, as pre-programmed algorithms were readily available from the BCT MATLAB toolbox [55]. Nevertheless, to avoid these issues, future studies could employ different methods of community detection, such as Bayesian-based methods [92].

Finally, a fifth limitation of the present study is its sole focus on functional topological complexity as quantified via information-theoretic measures. As stated in the introduction, the brain’s complexity can manifest temporally as well as topologically [16]. There are several metrics available to quantify the functional temporal complexity of the brain (e.g., Lempel–Ziv complexity, Hurst exponent, etc.) [15,16] and it is likely that these metrics are influenced in a very different way compared with how the factors explored here (volume conduction, source signal regularity, modularity) affect the brain’s functional topological complexity. For example, Lempel–Ziv complexity [93] reflects the irregularity of a data sequence and thus should be maximal for irregular EEG source signals and minimal for regular signals. Moreover, both temporal and topological complexity can be quantified by metrics other than those based in information theory. For instance, the brain’s functional topological complexity can also be quantified by modification of the Lempel–Ziv complexity [16], or by examining connectivity patterns among nodes and how this determines the spatial formation of node clusters [17]. The latter topological complexity metric follows an inverted-U relationship with modularity and the functional connectivity among brain network nodes [17]; how it would be affected by volume conduction and brain source regularity is unclear. Understanding how the temporal complexity of the brain is affected by such factors, and how they distinctively influence different brain complexity metrics, are topics for future research.

## 5. Conclusions

In conclusion, this study has shown that the previously observed discrepancy between the functional integration and topological complexity of cortical- versus scalp-level EEG signals primarily reflects the spatial mixing effects of volume conduction. This spatial mixing is also influenced by the regularity of the cortical EEG source signals, as the source- versus scalp-level differences in the functional integration–complexity relationship occur for irregular (aperiodic) cortical sources but not regular (periodic) cortical sources. This is because the spatial mixing of volume conduction temporally filters the volume-conducted EEG signals to become less irregular and more regular at the scalp. Furthermore, EEG source modularity mostly influences the functional integration–complexity relationship for regular EEG signals, with the relationship for irregular scalp EEG signals driven mainly by changes in EEG source regularity coupled with the effects of volume conduction. Future EEG studies of the brain’s functional organization must take these factors into account when interpreting the functional integration and topological complexity of the brain as measured at the scalp.

## Figures and Tables

**Table 1:** Observed EEG integration and complexity summary statistics.

	Observed Data		Surrogate Data	
Condition	I(X)	C_I_(X)	I(X)	C_I_(X)
Eyes closed	653.48 (15.91)	197.05 (13.90)	449.82 (30.90)	1231.24 (50.56)
	[646.23, 660.72]	[190.72, 203.38]	[435.75, 463.88]	[1208.23, 1254.25]
Eyes open	640.46 (11.94)	201.36 (14.89)	433.89 (29.35)	1249.21 (43.89)
	[635.02, 645.89]	[194.58, 208.13]	[420.53, 447.25]	[1229.23, 1269.19]

Note: Statistics shown are mean, *SD* (in parentheses), and 95% *CIs* (in brackets). All values are in units of bits.

**Table 2:** Observed EEG network parameters summary statistics.

	Condition	Q	PC	PI	N_M_	M_Size_	γ
Observed Data	Eyes closed	6.42 (0.44)	65.33 (2.13)	85.30 (1.15)	3.48 (0.20)	21.79 (1.34)	1.05 (0.01)
		[6.22, 6.62]	[64.36, 66.30]	[84.78, 85.83]	[3.39, 3.57]	[21.18, 22.39]	[1.05, 1.06]
	Eyes open	6.07 (0.43)	66.97 (1.52)	86.40 (0.87)	3.62 (0.13)	20.85 (0.89)	1.06 (0.01)
		[5.87, 6.26]	[66.28, 67.66]	[86.01, 86.80]	[3.56, 3.68]	[20.44, 21.25]	[1.05, 1.06]
Surrogate Data	Eyes closed	5.96 (0.29)	67.10 (1.14)	86.47 (0.66)	3.63 (0.12)	20.73 (0.70)	1.06 (0.01)
		[5.83, 6.10]	[66.58, 67.62]	[86.17, 86.76]	[3.58, 3.68]	[20.42, 21.05]	[1.05, 1.06]
	Eyes open	5.60 (0.22)	68.33 (0.83)	87.28 (0.44)	3.74 (0.09)	20.07 (0.52)	1.06 (0.01)
		[5.50, 5.70]	[67.95, 68.71]	[87.08, 87.48]	[3.70, 3.78]	[19.84, 20.31]	[1.05, 1.06]

Note: Statistics shown are mean, *SD* (in parentheses), and 95% *CIs* (in brackets). *Q*, *PC*, and *PI* are expressed as percentages of the original 0 to 1 range of these measures. *N_M_*, *M_Size_*, and *γ* are in dimensionless units.

**Table 3:** Across-participant correlations between EEG network parameters and EEG integration/complexity.

		ΔQ	ΔPC	ΔPI	ΔN_M_	ΔM_Size_	Δγ
ΔI(X)	*R*	0.72	−0.84	−0.89	−0.80	0.80	−0.73
	*p*	0.005	0.003	0.003	0.003	0.003	0.003
ΔC*_I_*(X)	*R*	−0.38	0.71	0.63	0.69	−0.70	0.73
	*p*	0.094	0.005	0.008	0.007	0.005	0.003

Note: All *p*-values are corrected for multiple comparisons (see Section 2.9.2: Parametric Statistics Section, above).

**Table 4:** Simulated EEG Network Parameters Results Summary.

Network Parameter	Simulation Level	Dipole Manipulation Phase 1	Dipole Manipulation Phase 2
N_M_ & M_Size_	EEG Dipole Sources	Total number of modules and module size fluctuated around a constant value across partitions.	Total number of modules decreased, and module size increased, across partitions.
	Scalp EEG Signals	Total number of modules and module size fluctuated around a constant value across partitions.	Total number of modules decreased, and module size increased across partitions.
PC & PI	EEG Dipole Sources	Participation coefficient/index decreased across partitions.	Participation coefficient/index decreased across partitions.
	Scalp EEG Signals	Participation coefficient/index decreased across partitions.	Participation coefficient/index decreased across partitions.
Q	EEG Dipole Sources	Modularity statistic increased across partitions.	Modularity statistic increased across partitions.
	Scalp EEG Signals	Modularity statistic increased across partitions.	Modularity statistic increased across partitions.
γ	EEG Dipole Sources	Increased from a minimum at Partition 1 to a maximum at Partition 9.	Decreased to a minimum at Partition 18.
	Scalp EEG Signals	Maintained a constant value across partitions.	Maintained a constant value before decreasing across Partitions 17 and 18.

**Table 5:** Across-participant correlations between simulated EEG network parameters and simulated EEG integration/complexity.

			Q	PC	PI	N_M_	M_Size_	γ
Source Level Simulation	I(X) Phases 1 and 2	*R*	0.96	−0.95	−0.97	−0.90	0.68	−0.79
		*p*	0.003	0.004	0.004	0.004	0.003	0.003
	C_I_(X) Phase 1	*R*	0.99	−0.99	−0.99	−0.49	0.48	0.94
		*p*	0.002	0.003	0.003	0.37	0.200	0.002
	C_I_(X) Phase 2	*R*	−0.75	0.98	0.95	0.99	−0.88	0.99
		*p*	0.060	0.002	0.002	0.002	0.001	0.001
Scalp Level Simulations	I(X) Phase 1 & 2	*R*	0.59	−0.92	−0.70	−0.94	0.89	−0.91
		*p*	0.013	0.002	0.006	0.002	0.002	0.001
	C_I_(X) Phase 1 & 2	*R*	−0.70	0.96	0.80	0.95	−0.91	0.93
		*p*	0.006	0.002	0.005	0.002	0.001	0.001

Note: All *p*-values are corrected for multiple comparisons (see Section 2.9.2 Parametric Statistics Section, above).

## Data Availability

The original data presented in the study are openly available in the Texas Data Repository at https://dataverse.tdl.org/dataverse/eeg_int_comp_r accessed on 28 June 2026.

## Supplementary Materials

The following supporting information can be downloaded at: https://www.mdpi.com/article/10.3390/brainsci16080796/s1, Figure S1: Unfiltered scalp-level EEG functional integration and complexity for the dipole simulations. Figure S2: Modular partitioning of the simulated alpha-range dipole moment sources. Figure S3: Modular partitioning of the scalp-level EEG signals after projecting the simulated alpha-range EEG dipole sources to the scalp.

## Abbreviations

The following abbreviations are used in this manuscript:

BCT	Brain Connectivity Toolbox
ciPLV	Corrected imaginary phase locking value
CIs	Confidence interval
CSD	Current source density
EEG	Electroencephalography
FFT	Fast Fourier transform
G-KNN	Geometric K-nearest neighbors
ICA	Independent components analysis
KSG	Kraskov–Stögbauer–Grassberger
PC	Participation coefficient
PI	Participation index
PLV	Phase locking value
SD	Standard deviation
SVD	Singular value decomposition
RTR	Run test for randomness
wPLI	Weighted phase lag index

## References

[1] Bullmore E., Sporns O. (2009). Complex brain networks: Graph theoretical analysis of structural and functional systems. Nat. Rev. Neurosci..

[2] Fair D.A., Cohen A.L., Power J.D., Dosenbach N.U.F., Church J.A., Miezin F.M., Schlaggar B.L., Petersen S.E. (2009). Functional brain networks develop from a “local to distributed” organization. PLoS Comput. Biol..

[3] Palma-Espinosa J., Orellana-Villota S., Coronel-Oliveros C., Maidana J.P., Orio P. (2025). The balance between integration and segregation drives network dynamics maximizing multistability and metastability. Sci. Rep..

[4] Tononi G., Edelman G.M., Sporns O. (1998). Complexity and coherency: Integrating information in the brain. Trends Cogn. Sci..

[5] Tononi G., McIntosh A.R., Russell D.P., Edelman G.M. (1998). Functional clustering: Identifying strongly interactive brain regions in neuroimaging data. Neuroimage.

[6] Tononi G., Sporns O., Edelman G.M. (1994). A measure for brain complexity: Relating functional segregation and integration in the nervous system. Proc. Natl. Acad. Sci. USA.

[7] Tononi G., Sporns O., Edelman G.M. (1996). A complexity measure for selective matching of signals by the brain. Proc. Natl. Acad. Sci. USA.

[8] Cohen J.R., D’Esposito M. (2016). The segregation and integration of distinct brain networks and their relationship to cognition. J. Neurosci..

[9] Hernández R.M., Ponce-Meza J.C., Saavedra-López M.A., Ugaz W.A.C., Chanduvi R.M., Monteza W.C. (2023). Brain complexity and psychiatric disorders. Iran. J. Psychiatry.

[10] Jang H., Mashour G.A., Hudetz A.G., Huang Z. (2024). Measuring the dynamic balance of integration and segregation underlying consciousness, anesthesia, and sleep in humans. Nat. Commun..

[11] Lord L.-D., Stevner A.B., Deco G., Kringelbach M.L. (2017). Understanding principles of integration and segregation using whole-brain computational connectomics: Implications for neuropsychiatric disorders. Philos. Trans. R. Soc. A Math. Phys. Eng. Sci..

[12] Sarasso S., Casali A.G., Casarotto S., Rosanova M., Sinigaglia C., Massimini M. (2021). Consciousness and complexity: A consilience of evidence. Neurosci. Conscious..

[13] Tononi G. (2004). An information integration theory of consciousness. BMC Neurosci..

[14] Wang R., Liu M., Cheng X., Wu Y., Hildebrandt A., Zhou C. (2021). Segregation, integration, and balance of large-scale resting brain networks configure different cognitive abilities. Proc. Natl. Acad. Sci. USA.

[15] Lau Z.J., Pham T., Annabel Chen S.H. (2022). Brain entropy, fractal dimensions and predictability: A review of complexity measures for EEG in healthy and neuropsychiatric populations. Eur. J. Neurosci..

[16] Varley T.F., Luppi A.I., Pappas I., Naci L., Adapa R., Owen A.M., Menon D.K., Stamatakis E.A. (2020). Consciousness & brain functional complexity in propofol anaesthesia. Sci. Rep..

[17] Zamora-López G., Chen Y., Deco G., Kringelbach M.L., Zhou C. (2016). Functional complexity emerging from anatomical constraints in the brain: The significance of network modularity and rich-clubs. Sci. Rep..

[18] Crutchfield J.P., Young K. (1989). Inferring statistical complexity. Physcal Rev. Lett..

[19] Gell-Mann M., Lloyd S. (1996). Information measures, effective complexity, and total information. Complexity.

[20] Michalowicz J.V., Nichols J.M., Bucholtz F. (2014). Handbook of Differential Entropy.

[21] Zurek W.H. (2023). Complexity, Entropy, & the Physics of Information Volume I.

[22] Zurek W.H. (2023). Complexity, Entropy, & the Physics of Information Volume II.

[23] Trujillo L.T. (2019). K-th nearest neighbor (KNN) entropy estimates of complexity and integration from ongoing and stimulus-evoked electroencephalographic (EEG) recordings of the human brain. Entropy.

[24] Trujillo L.T., Stanfield C.T., Vela R.D. (2017). The effect of electroencephalogram (EEG) reference choice on information-theoretic measures of the complexity and integration of EEG signals. Front. Neurosci..

[25] Nunez P.L., Srinivasan R. (2006). Electric Fields of the Brain: The Neurophysics of EEG.

[26] Dominicus L.S., Lodema D.Y., Oranje B., Zandstra M.G., Hermans A.P.C., Imhof L., Otte W.M., Hillebrand A., Ambrosen2 K., Stam C.J. (2025). Reliability and state-dependency of EEG connectivity, complexity and network characteristics. Sci. Rep..

[27] Fornito A.Z., Bullmore A. (2016). Fundamentals of Brain Network Analysis.

[28] van Cappellen van Walsum A.-M., Pijnenburg Y.A.L., Berendse H.W., van Dijk B.W., Knol D.L., Scheltens P., Stam C.J. (2003). A neural complexity measure applied to MEG data in Alzheimer’s disease. Clin. Neurophysiol..

[29] Bazanova O.M., Vernon D. (2014). Interpreting EEG alpha activity. Neurosci. Biobehav. Rev..

[30] Picton T.W., Bentin S., Berg P., Donchin E., Hillyard S.A., Johnson R., Miller G.A., Ritter W., Ruchkin D.S., Rugg M.D. (2000). Guidelines for using human event-related potentials to study cognition: Recording standards and publication criteria. Psychophysiology.

[31] Perrin F., Pernier J., Bertrand O., Echallier J.F. (1990). Corrigenda EEG 02274. Electroencephalogr. Clin. Neurophysiol..

[32] Perrin F., Pernier J., Bertrand O., Giard M.H., Echallier J.F. (1987). Mapping of scalp potentials by surface spline interpolation. Electroencephalogr. Clin. Neurophysiol..

[33] Law S.K., Nunez P.L., Wijesinghe R.S. (1993). High resolution EEG using spline generated surface laplacians on spherical and ellipsoidal surfaces. IEEE Trans. Biomed. Eng..

[34] Zhang Y., Chen Z.S. (2025). Harnessing electroencephalography connectomes for cognitive and clinical neuroscience. Nat. Biomed. Eng..

[35] Kayser J. (2009). Current Source Density (CSD) Interpolation Using Spherical Splines—CSD Toolbox (Version 1.1).

[36] Kayser J., Tenke C.E. (2006). Principal components analysis of Laplacian waveforms as a generic method for identifying ERP generator patterns: I. Evaluation with auditory oddball tasks. Clin. Neurophysiol..

[37] Kayser J., Tenke C.E. (2006). Principal components analysis of Laplacian waveforms as a generic method for identifying ERP generator patterns: II. Adequacy of low-density estimates. Clin. Neurophysiol..

[38] Klimesch W. (1999). EEG alpha and theta oscillations reflect cognitive and memory performance: A review and analysis. Brain Res. Rev..

[39] Wen H.L. (2016). Separating fractal and oscillatory components in the power spectrum of neurophysiological signal. Brain Topogr..

[40] Addison P.S. (2017). The Illustrated Wavelet Transform Handbook: Introductory Theory and Applications in Science, Engineering, Medicine, and Finance.

[41] Delorme A., Makeig S. (2004). EEGLAB: An open source toolbox for analysis of single-trial EEG dynamics including independent component analysis. J. Neurosci. Methods.

[42] Feige B., Scheffler K., Esposito F., Di Salle F., Hennig J., Seifritz E. (2005). Cortical and subcortical correlates of electroencephalographic alpha rhythm modulation. J. Neurophysiol..

[43] Srinivasan R., Winter W.R., Nunez P.L. (2006). Source analysis of EEG oscillations using high-resolution EEG and MEG. Prog. Brain Res..

[44] Jensen O., Hesse C., Hansen P.C., Kringelbach M.L., Salmelin R. (2010). Estimating distributed representations of evoked responses and oscillatory brain activity. MEG: An Introduction to Methods.

[45] Cuffin B.N., Cohen D. (1979). Comparison of the magnetoencephalogram and electroencephalogram. Electroencephalogr. Clin. Neurophysiol..

[46] Mosher J.C., Spencer M.E., Leahy R.M., Lewis P.S. (1993). Error bounds for EEG and MEG source localization. Electroencephalogr. Clin. Neurophysiol..

[47] Tenke C.E., Kayser J. (2015). Surface Laplacians (SL) and phase properties of EEG rhythms: Simulated generators in a volume-conduction model. Int. J. Psychophysiol..

[48] Gibbons J.D., Chakraborti S. (2021). Nonparametric Statistical Inference.

[49] Lord W.M., Sun J., Bollt E.M. (2018). Geometric k-nearest neighbor estimation of entropy and mutual information. Chaos.

[50] Charsyńska A., Gambin A. (2015). Improvement of the k-NN entropy estimator with applications in systems biology. Entropy.

[51] Kraskov A., Stögbauer H., Grassberger P. (2004). Estimating mutual information. Phys. Rev. E.

[52] Gao S., Ver Steeg G., Galstyan A. Efficient estimation of mutual information for strongly dependent variables. Proceedings of the Eighteenth International Conference on Artificial Intelligence and Statistics.

[53] von Luxburg U.A. Density estimation from unweighted k-nearest neighbor graphs: A roadmap. Proceedings of the 27th Annual Conference on Neural Information Processing Systems (NIPS 2013).

[54] Khan S., Bandyopadhyay S., Ganguly A.R., Saigal S., Erickson D.J., Protopopescu V., Ostrouchov G. (2007). Relative performance of mutual information estimation methods for quantifying the dependence among short and noisy data. Phys. Rev. E.

[55] Rubinov M., Sporns O. (2010). Complex network measures of brain connectivity: Uses and interpretations. Neuroimage.

[56] Bruña R.M., Pereda F. (2018). Phase locking value revisited: Teaching new tricks to an old dog. J. Neural Eng..

[57] Vinck M.O., van Wingerden R., Battaglia M., Pennartz F. (2011). An improved index of phase-synchronization for electrophysiological data in the presence of volume-conduction, noise and sample-size. Neuroimage.

[58] Colclough G.L.W., Tewarie M.W., Brookes P.K., Quinn M.J., Smith A.J. (2016). How reliable are MEG resting-state connectivity metrics?. Neuroimage.

[59] Garcés P.M.-B., Maestú M.C. (2016). Quantifying the test-retest reliability of magnetoencephalography resting-state functional connectivity. Brain Connect..

[60] Blondel V.D.G., Lambiotte J.-L., Lefebvre R. (2008). Fast unfolding of communities in large networks. J. Stat. Mech. Theory Exp..

[61] Newman M.E.J. (2004). Analysis of weighted networks. Phys. Rev. E.

[62] Reichardt J.B. (2006). Statistical mechanics of community detection. Phys. Rev. E.

[63] Rubinov M.S. (2011). Weight-conserving characterization of complex functional brain networks. Neuroimage.

[64] Newman M.E.J.G. (2004). Finding and evaluating community structure in networks. Phys. Rev. E.

[65] Good B.H.D.M., Clauset Y.-A. (2010). Performance of modularity maximization in practical contexts. Phys. Rev. E.

[66] Newman M.E.J. (2016). Equivalence between modularity optimization and maximum likelihood methods for community detection. Phys. Rev. E.

[67] Guimerà R.A. (2005). Functional cartography of complex metabolic networks. Nature.

[68] Klimm F.B.-H., Wessel J., Kurths N., Zamora-López J. (2014). Individual node’s contribution to the mesoscale of complex networks. New J. Phys..

[69] Lachaux J.-P., Rodriguez E., Le Van Quyen M., Lutz A., Martinerie J., Varela F.J. (2000). Studying single-trials of phase synchronous activity in the brain. Int. J. Bifurcat. Chaos.

[70] Lachaux J.-P., Rodriguez E., Martinerie J., Varela F.J. (1999). Measuring phase synchrony in brain signals. Hum. Brain Mapp..

[71] Nunez P.L., Srinivasan R., Westdorp A.F., Wijesinghe R.S., Tucker D.M., Silberstein R.B., Cadusch P.J. (1997). EEG coherency I: Statistics, reference electrode, volume conduction, Laplacians, cortical imaging, and interpretation at multiple scales. Electroencephalogr. Clin. Neurophysiol..

[72] Shahbazi F., Ewald A., Ziehe A., Nolte G., Supek S., Sušac A. (2010). Constructing surrogate data to control for artifacts of volume conduction for functional connectivity measures. 17th International Conference on Biomagnetism Advances in Biomagnetism—Biomag2010. IFMBE Proceedings.

[73] Theiler J., Eubank S., Longtin A., Galdrikian B., Farmer J.D. (1992). Testing for nonlinearity in time series: The method of surrogate data. Phys. D.

[74] Lee T.-W., Girolami M., Sejnowski T.J. (1999). Independent component analysis using an extended infomax algorithm for mixed sub-gaussian and super-gaussian sources. Neural Comput..

[75] Nichols T.E., Holmes A.P. (2002). Nonparametric permutation tests for functional neuroimaging: A primer with examples. Hum. Brain Mapp..

[76] Crosse M.J.F., Molholm J.J. (2024). PERMUTOOLS: A Matlab Package for Multivariate Permutation Testing. arXiv.

[77] Holm S. (1979). A simple sequentially rejective multiple test procedure. Scand. J. Stat..

[78] Niedermeyer E., Niedermeyer E.L.D., Silva F. (1999). The normal EEG of the waking adult. Electroencephalography: Basic Principles, Clinical Applications and Related Fields.

[79] Witkowski S., Trujillo L.T., Sherman S.M., Carter P., Matthews M.D., Schnyer D.M. (2015). An examination of the association between chronic sleep restriction and electrocortical arousal in college students. Clin. Neurophysiol..

[80] Norwich K.H. (1993). Information, Sensation, and Perception.

[81] Pernier J., Perrin F., Bertrand O. (1988). Scalp current density fields: Concept and properties. Electroencephalogr. Clin. Neurophysiol..

[82] Dien J. (1998). Issues in the application of the average reference: Review, critiques, and recommendations. Behav. Res. Methods Instrum. Comput..

[83] Barry R.J., De Blasio F.M. (2017). EEG differences between eyes-closed and eyes-open resting remain in healthy ageing. Biol. Psychol..

[84] Gómez-Ramírez J., Freedman S., Mateos D., Velázquez J.L.P., Valiante T.A. (2017). Exploring the alpha desynchronization hypothesis in resting state networks with intracranial electroencephalography and wiring cost estimates. Sci. Rep..

[85] Krukow P., Rodríguez-González V., Kopiś-Posiej N., Gómez C., Poza J. (2024). Tracking EEG network dynamics through transitions between eyes-closed, eyes-open, and task states. Sci. Rep..

[86] Tan B., Kong X., Yang P., Jin Z., Li L. (2013). The difference of brain functional connectivity between eyes-closed and eyes-open using graph theoretical analysis. Comput. Math. Methods Med..

[87] Buzáki G., Başar E., Bullock T.H. (1992). Network properties of the thalamic clock: Role of oscillatory behavior in mood disorders. Induced Rhythms in the Brain.

[88] Choi K.-M., Kim J.-Y., Kim Y.-W., Han J.-W., Im C.-H., Lee S.-H. (2021). Comparative analysis of default mode networks in major psychiatric disorders using resting-state EEG. Sci. Rep..

[89] Groppe D.M., Bickel S., Keller C.J., Jain S.K., Hwang S.T., Harden C., Mehta A.D. (2013). Dominant frequencies of resting human brain activity as measured by the electrocorticogram. Neuroimage.

[90] Mantini D., Perrucci M.G., Del Gratta C., Romani G.L., Corbetta M. (2007). Electrophysiological signatures of resting state networks in the human brain. Proc. Natl. Acad. Sci. USA.

[91] Démas J., Bourguignon M., Périvier M., De Tiège X., Dinomais M., Van Bogaert P. (2020). Mu rhythm: State of the art with special focus on cerebral palsy. Ann. Phys. Rehabil. Med..

[92] Mørup M., Schmidt M.N. (2012). Bayesian community detection. Neural Comput..

[93] Lempel A., Ziv J. (1976). On the complexity of finite sequences. IEEE Trans. Inf. Theory.

