# Supplementary material for: The Relationship Between the Functional Integration and Topological Complexity of Human Electroencephalographic (EEG) Signals: Three Moderating Factors
Source: Brain Sci. 2026 Jul 28;16(8):796. doi: 10.3390/brainsci16080796 (PMC13510657; doi:10.3390/brainsci16080796)
Supplement: Supplementary file 1 [file brainsci-16-00796-s001.zip › brainsci-4437798-supplementary.pdf]

Supplementary Materials for "The Relationship Between the Functional Integration and Topological Complexity of Human Electroencephalographic (EEG) Signals: Three Moderating Factors"

By Logan T. Trujillo

Published in Brain Sciences (2026)

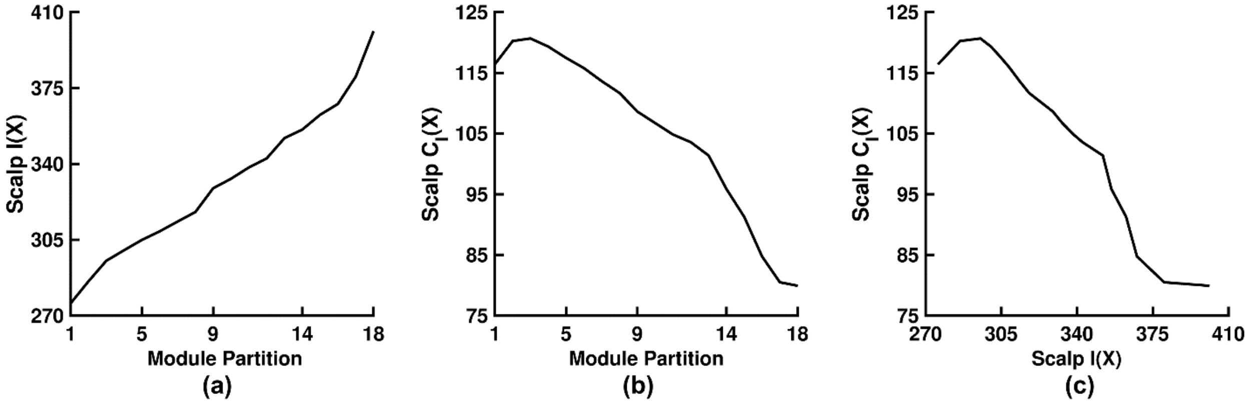

Figure S1. Unfiltered scalp-level EEG functional integration and complexity for the dipole simulations. (a) Integration *I(X)* as a function of Phase 1 and 2 Module Partitions for simulated whole-scalp EEG resulting from volume-conducted activity of all dipole sources (occipital and background dipoles). (b) Interaction complexity $C_I(X)$ as a function of Phase 1 and 2 Module Partitions for simulated scalp EEG. (c) Interaction complexity $C_I(X)$ as a function of integration *I(X)* for simulated scalp EEG. All values are in units of bits. Data points reflect averages across 432 simulated trials; the standard deviations of simulated *I(X)* and $C_I(X)$ are too small to be displayed but ranged from 0.03 to 1.10 bits.

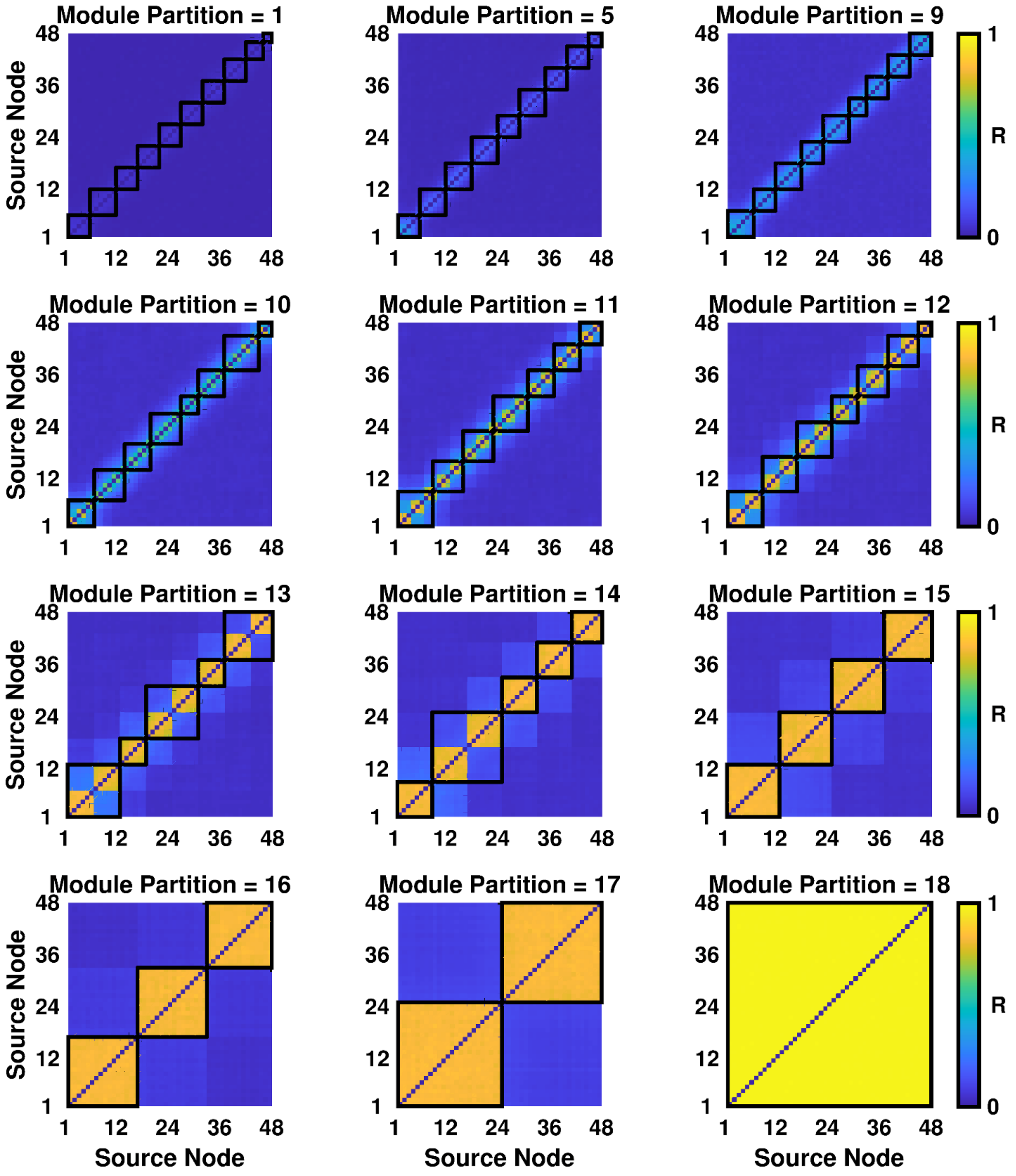

Figure S2. Modular partitioning of the simulated alpha-range dipole moment sources. Each subplot depicts the adjacency matrix for each module partition, with the colors in each plot reflecting the linear correlations between each non-identical source dipole node pair. The overlaid black boxes reflect the module partitions as estimated by the community detection algorithm (see main text). Note that as the adjacency matrix plots for the Phase 1 module partitions were very similar, only three representative Phase 1 module partitions (Module Partitions 1, 5, and 9) are shown.

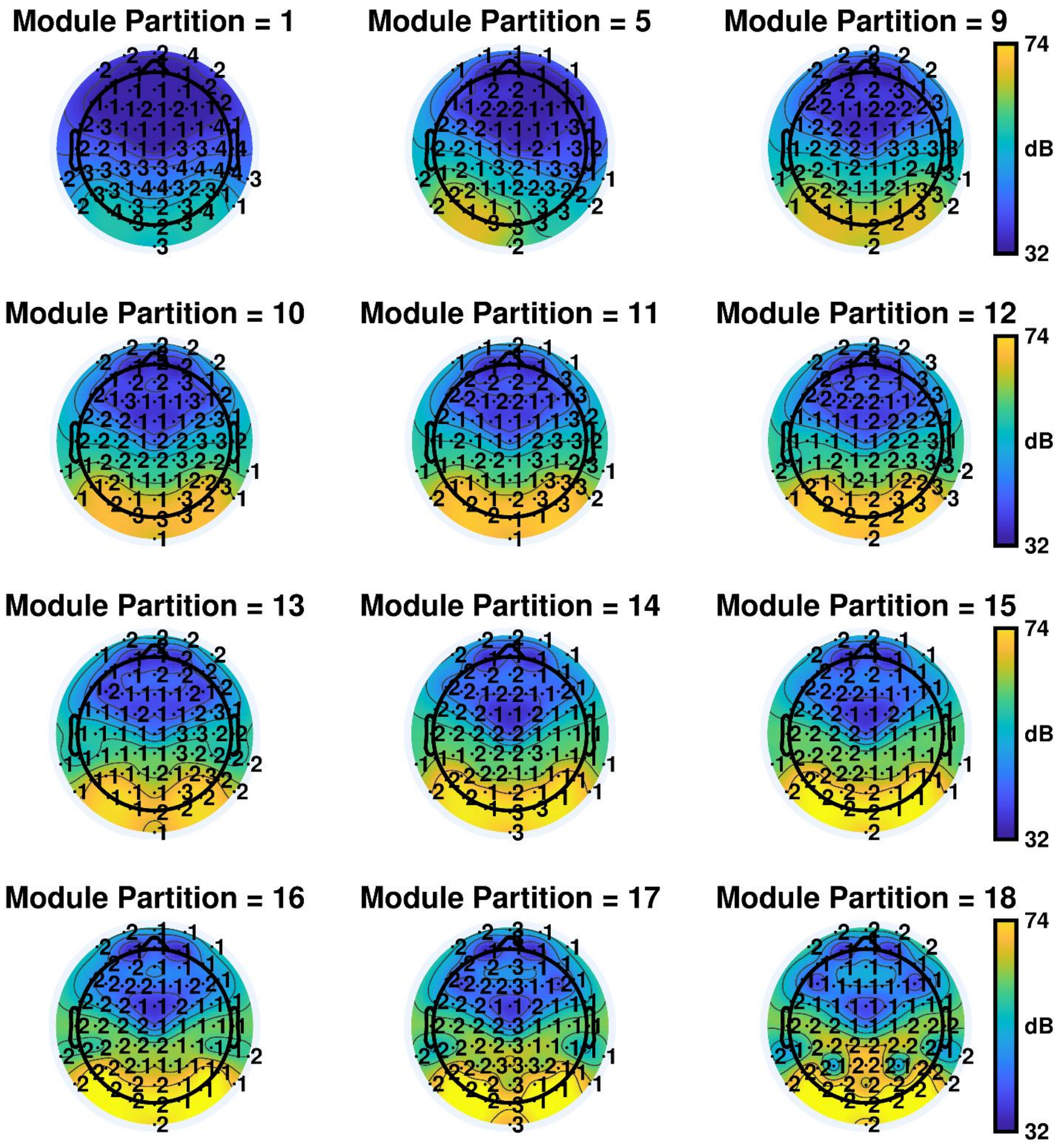

Figure S3. Modular partitioning of the scalp-level EEG signals after projecting the simulated alpha-range EEG dipole sources to the scalp. Note that these results now also include a contribution from the random background sources present in the EEG dipole model. Each number indicates the representative module label (e.g., Module 1, Module 2, etc.) for each electrode site. These values were determined by taking the cross-trial mode of module label numbers returned by the community detection algorithm (see main text) at each electrode site for each trial. Head map colors display the scalp topography of the average alpha-range *PSD* for each partition. Note that the pattern of estimated modularity roughly follows the variation of *PSD* across the scalp. Also note that as the scalp-map plots for the Phase 1 module partitions were very similar, plots for only three representative Phase 1 module partitions (Module Partitions 1, 5, and 9) are shown.
